# Developing a best practice framework for musculoskeletal outpatient physiotherapy delegation: the MOPeD mixed-methods research study protocol

**DOI:** 10.1136/bmjopen-2023-072989

**Published:** 2023-03-17

**Authors:** Panos Sarigiovannis, Nadine E Foster, Sue Jowett, Benjamin Saunders

**Affiliations:** 1 Primary Care Centre Versus Arthritis, School of Medicine, Keele University, Keele, UK; 2 North Integrated Musculoskeletal Service, Midlands Partnership NHS Foundation Trust, Stafford, UK; 3 Surgical Treatment and Rehabilitation Service (STARS) Education and Research Alliance, The University of Queensland and Metro North Health, Herston, Brisbane, Australia; 4 Health Economics Unit, Institute of Applied Health Research, Public Health Building, University of Birmingham, Birmingham, UK

**Keywords:** Adult orthopaedics, Musculoskeletal disorders, Health & safety

## Abstract

**Introduction:**

Physiotherapy assistants/support workers are an important part of the physiotherapy workforce in the UK. Many of them work in National Health Service (NHS) physiotherapy outpatient services treating patients with musculoskeletal (MSK) conditions. In many services, they take responsibility, under professional supervision, for types of clinical work traditionally undertaken by physiotherapists such as leading exercise classes and treating individual patients. Nevertheless, their role(s) are relatively undefined and as such, there is considerable variation in the duties and tasks they undertake. This study aims to design a framework of ‘best practice’ in delegation to guide the work of clinicians in NHS physiotherapy MSK services and facilitate standardisation of practice to ensure that patients receive safe and effective treatment by the most appropriate person.

**Methods and analysis:**

This mixed-methods study will be conducted in four stages. In stage 1, a focused ethnography in two MSK outpatient physiotherapy services will explore how the current use of delegation is informed by the culture within the clinical setting as well as views, attitudes about, and experiences of, delegation among clinicians, managers and patients. In stage 2a, nominal group technique will be used with three separate groups (physiotherapists/physiotherapy assistants/support workers, managers) to reach a consensus about what components should be included in a best practice framework of delegation. In stage 2b, a discrete choice experiment will elicit patients’ preferences between care from physiotherapists and physiotherapy assistants/support workers within MSK physiotherapy services. In the final stage, the results of all previous stages will be triangulated to inform the development of a best practice delegation framework for future testing and use within NHS MSK outpatient physiotherapy services.

**Ethics and dissemination:**

Ethical approval has been granted by the South West-Frenchay Research Ethics Committee. The findings will be disseminated in peer-reviewed journals, conference presentations, the lay press and social media.

**STRENGTHS AND LIMITATIONS OF THIS STUDY**

- A mixed-methods study, which includes qualitative and quantitative data and multiple research approaches, will enable a more comprehensive understanding of the issues that affect delegation in the musculoskeletal (MSK) physiotherapy setting.
- The design of a framework of best practice in delegation to guide the work of physiotherapy assistants/support workers in the MSK physiotherapy setting, underpinned by patients’ preferences and clinicians’ consensus, may facilitate successful implementation in clinical practice.
- The focused ethnographic study is being conducted across two National Health Service (NHS) Trusts and the discrete choice experiment across one NHS Trust; therefore, the results may not be representative of the NHS more broadly.
- Patients and clinicians have been involved in designing the research and they will continue to be involved in all stages of the study.

## Introduction

Musculoskeletal (MSK) conditions such as low back pain and osteoarthritis affect one in four people globally, are increasingly common with age, are the leading cause of pain and disability in the UK and the second leading cause of sickness absence from work.^1^ Patients with MSK conditions are the largest patient population group treated by physiotherapists.^2^ Patients are assessed by physiotherapists and if they need follow-up treatments, they are usually treated by either a physiotherapist or a physiotherapy support worker. Physiotherapy support workers are non-registered staff who work alongside physiotherapists to provide delegated interventions and responsibilities. They may also be known as physiotherapy assistants, rehabilitation assistants, technical instructors or physiotherapy technicians. There are approximately 9000 physiotherapy assistants/support workers in the UK, forming 15% of the total physiotherapy workforce and a large proportion of them work in the National Health Service (NHS). In many services, they take responsibility, under professional supervision, for certain types of clinical work traditionally undertaken by qualified physiotherapists, such as leading exercise classes and treating individual patients. However, their role(s) are relatively undefined and as such, there is considerable variation in the duties and tasks that they undertake.^3^ National guidance from the Chartered Society of Physiotherapy (CSP) (CSP is the professional, educational and trade union body for the UK’s chartered physiotherapists, physiotherapy students and support workers) about delegation of tasks to physiotherapy assistants/support workers largely leaves decision-making to the individual physiotherapist, their judgement of the task and their assessment of the competence of the physiotherapy assistant/support worker.^4^ As a result, in some physiotherapy services, physiotherapy assistants/support workers have a predominantly clinical role, whereas in others, they fulfil primarily an administrative role such as data inputting and booking appointments. This latter situation leads to physiotherapy assistants/support workers not being able to use their clinical skills, experiencing job dissatisfaction, as well as to unjustified variation in care and clinical services provided to patients.^3^ Results from a recent systematic review, which explored the clinical and cost-effectiveness and perceptions of delegation by allied health professionals to allied health assistants internationally,^5^ highlighted that delegation is not standardised within physiotherapy and that there are clear knowledge gaps regarding delegation by physiotherapists in current practice. These relate to the clinical and cost-effectiveness of delegation as well as patients’ preferences, experiences of and attitudes about delegation. This study aims to design a framework of best practice in delegation to guide the work of physiotherapy assistants/support workers in NHS physiotherapy MSK services and facilitate standardisation of practice to ensure that patients receive safe and effective treatment by the most appropriate person.

## Rationale

The NHS Long Term Plan includes a commitment to narrow health inequalities and address unwarranted variation in care.^6^ A framework of best practice for delegation within the MSK setting could facilitate standardisation of delegation in physiotherapy and therefore, minimise unwarranted variation in the provision of physiotherapy services. Appropriate use of physiotherapy assistants/support workers could reduce healthcare costs either directly or indirectly since it could release capacity for physiotherapists to treat patient cases that are more complex or to be the first point of contact for some patients, in place of a general practitioner or a consultant doctor. Most importantly, patients would see the right staff with the right skills at the right time, which could potentially optimise clinical outcomes and patient satisfaction. Since staffing costs usually account for between 60% and 80% of operating costs in healthcare services, determining the ‘right’ combination of staff with the right skills is a critical component of successful and efficient healthcare delivery.^7 8^ The findings of this research will guide workforce planning in MSK physiotherapy services and identify future training needs. Finally, the best practice delegation framework will underpin a future research study, which will assess the clinical and cost-effectiveness of delegation using this framework in clinical settings.

## Theoretical framework

An exploratory sequential mixed-methods design will be used. This is characterised by an initial qualitative phase of data collection and analysis followed by a quantitative phase. It also includes a final phase of integration or linking the data from the two separate strands of data. The conceptual framework in the exploratory design will be inductively developed in the initial phase of the study where qualitative data results may lead to a theoretical model.^9^ One of the strengths of the exploratory sequential design is that the researcher can produce a new instrument or a framework as one of the potential products of the research process.^9^

## Research question/aim(s)

The overall research question is:

‘What should a ‘best practice’ framework of delegation incorporate and how can this be operationalised to guide utilisation of physiotherapy assistants/support workers in NHS MSK outpatient physiotherapy services?’

### Objectives

The specific objectives are to:

1. Explore how the use of delegation is informed by the culture within the clinical setting as well as perceptions of, and attitudes about, delegation among physiotherapists, physiotherapy assistants/support workers, physiotherapy managers and patients.
2. Reach a consensus between physiotherapists, physiotherapy assistants/support workers and physiotherapy managers about what constitutes ‘best practice’ and what components should be included in a best practice framework of delegation in NHS MSK outpatient physiotherapy services.
3. Explore patients’ preferences in relation to delegation in NHS MSK outpatient physiotherapy services and estimate specific trade-offs patients are willing to make in treatment choices when they are treated by physiotherapy assistants/support workers.
4. Develop a best practice delegation framework, which can be tested in future research and used within NHS MSK outpatient physiotherapy services.

**Figure 1:**
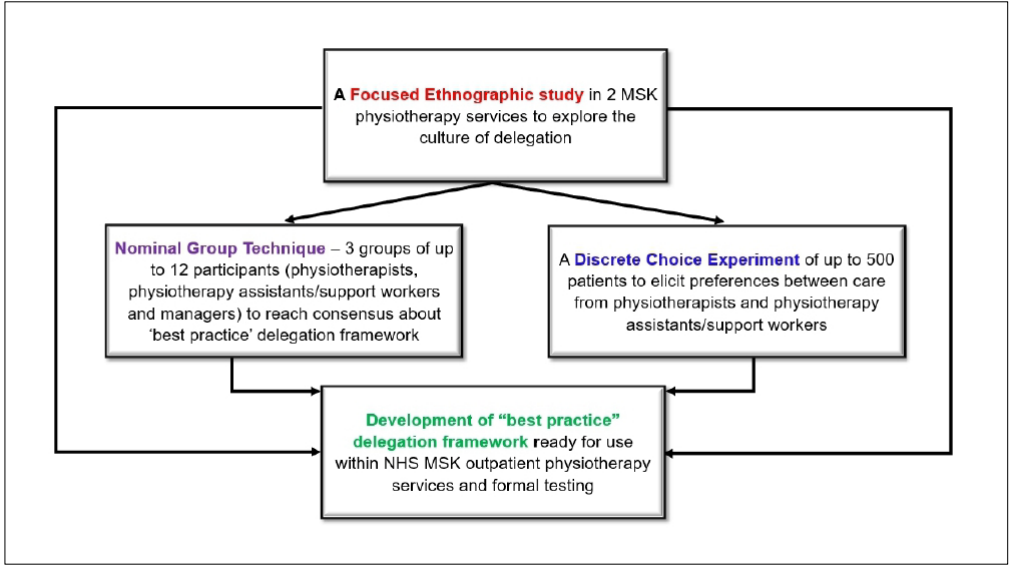
Outline of the MOPeD Study. MSK, musculoskeletal; NHS, National Health Service.

An outline of the MOPeD Study is shown in figure 1.

## Methods and analysis

### Eligibility criteria

The eligibility criteria for each stage of the MOPeD Study are shown in table 1.

**Table 1:** Eligibility criteria for each stage

MOPeD Study eligibility criteria	
Stage 1: focused ethnography	Adult patients (18 years old or older) with an MSK condition who attend physiotherapy appointments at participating clinics during the period of the study. Physiotherapists, physiotherapy assistants/support workers and physiotherapy managers working in participating MSK outpatient physiotherapy services.
Stage 2a: consensus study	Experienced physiotherapists (Agenda for Change* band 6 or higher) working in the UK NHS in the treatment of MSK conditions. Physiotherapy assistants/support workers working in the UK NHS, treating patients with MSK conditions. Physiotherapy service managers working in the UK NHS outpatient MSK physiotherapy services.
Stage 2b: discrete choice experiment	Adult patients (18 years old or older) who have been offered a follow-up physiotherapy appointment and/or completed a course of physiotherapy treatment for an MSK condition in one of the MSK outpatient physiotherapy clinics within the participating NHS Trust.

*Physiotherapists working in the NHS are employed under the Agenda for Change grading and pay system where higher bandings are associated with higher qualifications and pay. Newly qualified physiotherapists work in band 5 positions, while physiotherapists who have completed their junior rotations are in band 6 positions.

MSK, musculoskeletal; NHS, National Health Service.

### Stage 1

#### Design: a focused ethnographic study

A focused ethnography will be conducted in two MSK outpatient physiotherapy services. Focused ethnography explores a specific issue, situation or problem within a specific context.^10 11^ It is very suitable for healthcare research as it provides an efficient way to capture in-depth data on a specific topic of importance to individual clinicians or clinical specialties, and to determine ways to improve care and care processes.^12^ This study has a clear focus on delegation, in a specific clinical setting and the research team has prior experience of using and organising clinical delegation. Therefore, focused ethnography will enable the researchers to explore how the use of delegation is informed by the culture of two different clinical settings, which will allow for the development of an in-depth and comparative understanding about how delegation is currently being used, and the factors that influence delegation to physiotherapy assistants/support workers at an individual, collective and broader organisation level. It will also allow the researchers to better understand attitudes about, and experiences of, delegation among physiotherapists, physiotherapy assistants/support workers, physiotherapy managers and patients.

#### Data collection

##### Stage 1/focused ethnography

A purposive sampling design has been selected as two physiotherapy services that are known to use delegation successfully (positive deviance approach) will be observed. These were chosen in collaboration with the appropriate professional advisor from the CSP who currently leads the support workers’ workstream, taking into consideration different indicators such as continuous use of physiotherapy assistants/support workers in treating patients with MSK conditions and dissemination of clinical practice. Within stage 1, criterion sampling will be used as the purposive sampling design for the selection of patients who will be invited to participate in the observations and/or interviews as only patients who have been treated by a physiotherapist and a physiotherapy assistant/support worker will be invited.

The researcher will use participant observation in two clinical sites focusing on current delegation practice, semistructured interviews with physiotherapists, physiotherapy assistants/support workers, managers and patients, as well as a review of physiotherapy treatment records and clinical site records such as policies, job descriptions and delegation training records to gain a rich and comprehensive understanding of how delegation works in the observed setting. Whenever appropriate, similarities and differences across the different types of data will be explored. Field observation will involve observing a range of activities including clinical sessions (one to one or groups) that physiotherapy assistants/support workers deliver independently or participate in, supervision sessions, training sessions and team meetings. Field notes will not only include information about observed events but also the researcher’s personal reflections and interpretation of events.^13^

All physiotherapy assistants/support workers who work in the observed clinical setting, the physiotherapists who delegate clinical tasks to them, the operational manager(s)/team leader(s) and patients will be invited to participate in the interviews. Up to 12 patients and 12 clinicians (physiotherapists, physiotherapy assistants/support workers, manager/s) will be interviewed at each site (ie, up to 24 interviews in total per site). A final decision on sample size will be made once data collection and analyses are ongoing, based on data saturation.^14^

The interview topic guides have been developed based on the evidence from a systematic review conducted by Sarigiovannis *et al*.^5^ An example of the interview topic guides is included in online supplemental appendix 1. The interviews will give the researcher the opportunity to ask physiotherapists, physiotherapy assistants/support workers, managers and patients for elaborations about specific topics, explanations of observed events and clarification of ambiguities.^15^ Questions will cover topics such as how the tasks were delegated, how patients feel about the delegation of clinical tasks, etc. The questions have been reviewed by the study’s Patient and Public Involvement and Engagement (PPIE) group and Clinical Advisory Group to ensure acceptability of the questions and ease of understanding. The examination of relevant documents such as patients’ physiotherapy treatment records and relevant policies will help validate the data from observations and interviews.

10.1136/bmjopen-2023-072989.supp1 Supplementary data

#### Data analysis

Data analysis will be based on the approach described by Roper and Shapira.^15^ Analytical steps include coding field notes and interviews, sorting to identify patterns for descriptive labels, identifying outliers or cases that do not ‘fit’ with the rest of the findings, generalising in relation to existing concepts and theories as well as the ideas and insights the researcher has about the data, including their reflective field notes.^15^ The analytical steps will be focused on answering specific problem-orientated research questions and the creation of concrete recommendations. The qualitative data analysis software QSR NVivo will be used to facilitate analysis. Data collection for stage 1 started in March 2022. The data analysis will be completed by June 2023.

### Stage 2a

#### Design: a nominal group technique approach

A consensus study will be carried out, using nominal group technique (NGT), a systematic approach to building a consensus using a structured set of stages. A convenience sample of physiotherapists, physiotherapy assistants/support workers and physiotherapy managers who work in NHS MSK physiotherapy services will be recruited through the CSP’s professional networks and through social media (Facebook, Twitter and LinkedIn) accounts of the authors and their networks. Three separate role-specific NGT working groups will be convened with: (1) physiotherapists, (2) managers and (3) physiotherapy assistants/support workers with the aim of reaching agreement about what a ‘best practice’ delegation framework should include. Each participant group will take part in a single meeting lasting approximately 2 hours. Two facilitators will lead each meeting. They will use a ‘pre-elicitation technique’ to inform the participants’ decision-making.^16^ Specifically, prior to the meeting, participants will receive a summary of existing evidence based on the findings from a systematic review conducted by Sarigiovannis *et al*.^5^ The participants will initially be asked to silently generate ideas about what should be included in a delegation framework for use within the MSK physiotherapy setting, which will then be shared with the group one by one. Each shared item will then be individually rated by all participants. Following this, the results of these ratings will be discussed within the group, and then they will be rerated individually by participants, who will be given the opportunity to amend their scores in light of group discussions.

The rating process will be explained step by step and participants will be given enough time for voting.^17^ Each item will be rated on a 7-point Likert scale by individual participants and a mean rating calculated. The threshold for a consensus will be set at ≥70%, that is, ≥4.9 on the 7-point Likert scale across the group.^18 19^ The data from the three groups will be combined into one complete sample to identify the most highly prioritised items.^20^ This includes calculating the mean scores for the raw data, the mean scores for the themes and the frequency of themes (ie, how many times a theme appeared in the top five and how often an idea was raised and coded under the same theme). All items will be categorised using a modified thematic analysis approach, which will include generating initial themes from the collated data and refining themes. The items will be rated using an online voting platform called Mentimeter (https://www.mentimeter.com/), which participants will access on their smartphones or laptops. The issues discussed during the meeting will not include any topics that might be sensitive, embarrassing or upsetting. The facilitators will keep notes during the consensus meetings. A list of all items that reached a consensus categorised into themes will be the final output from the consensus study.

### Stage 2b

#### Design: a discrete choice experiment

Discrete choice experiments (DCEs) are an attribute-based survey method for measuring benefits (utility). Within healthcare, the technique is applied to address a wide range of issues in the delivery of healthcare including measuring and valuing attributes of a healthcare service and identifying the factors that influence choices and decisions of patients, the public and healthcare professionals.^21^ They are based on the assumption that a service can be described by its characteristics or attributes, and the extent to which an individual values the service depends on the levels of these characteristics.^21^ In a DCE, respondents are asked to choose between two and more choice sets.

A DCE will be designed based on the data from the ethnography as well as further input from the study’s Clinical Advisory Group and PPIE group. The DCE will be conducted to elicit patients’ preferences about the use of delegation to physiotherapy assistants/support workers within MSK physiotherapy services. Convenience sampling will be used during this stage to invite patients who have been offered a follow-up physiotherapy appointment and/or completed a course of physiotherapy treatment programme in one of the MSK outpatient physiotherapy services within the participating NHS Trust. Patients will be invited by the treating clinicians when patients attend their follow-up physiotherapy appointment/are discharged. The minimum sample size needed for the DCE depends on the specific hypotheses to be tested.^22^ Therefore, the power of the DCE will be calculated when the questionnaire is finalised. It is expected that the number of participants will not exceed 500. This number is feasible since approximately 1500 patients are discharged per month within the Trust.

#### Data collection

Development of the attributes and levels will be undertaken using the appropriate findings from a systematic review on delegation by allied health professionals to support workers^5^; the ethnographic data from stage 1 of the study and further input from the study’s PPIE group.^23^ A recent systematic review of DCEs in healthcare reported that most studies included four to nine attributes, and among them, four to five were the modal category.^24^ The intention is to select between five and seven attributes. The Ngene design software will be used to create the choice sets. In addition to the DCE, the survey will include items to elicit sociodemographic information such as age, gender and employment status, as well as experience of being treated by a physiotherapy assistant/support worker. These seem to play an important role in predicting healthcare choices.^25^

The DCE survey will be offered in an online format, completed in physiotherapy clinics using tablet devices. The online version will be saved on a secure server. The understanding of the attributes and levels will be pretested among members of the PPIE group. The survey will then be piloted with a small sample of patients with MSK conditions consulting physiotherapy services. This will include testing respondent understanding of the different choices offered, generation and testing of appropriateness and understanding of attributes/levels, task complexity, length, timing and likely response rates.^26 27^

#### Data analysis

Data analysis will be completed using the STATA software. Although preference heterogeneity has long been accounted for in the analysis of DCEs by interacting design attributes with sociodemographic characteristics, evidence suggests that this approach only partially accounts for the differences in preference embodied in the data.^28^ Research has shown that DCE models that take into account both preference heterogeneity and differences in the error variance of choices (scale heterogeneity) are better to predict choices mimicking real-world decisions.^23^ Therefore, selecting the appropriate model for data analysis is important. The choice of the model will be finalised following discussions with DCE experts. The results will help the researchers understand what influences patients’ preferences by estimating the value patients place on key attributes and associated attribute levels. The researchers will estimate specific trade-offs that patients who are treated in NHS MSK outpatient physiotherapy services are willing to make in treatment choices, specifically in relation to being treated by physiotherapy assistants/support workers.

### Stage 3

#### Design and analysis: development of a best practice framework of delegation

The results from stages 1, 2a and 2b will be triangulated to inform the design of a best practice delegation framework for NHS MSK outpatient physiotherapy services. Triangulation will involve listing the findings from each stage of the study and consider where findings from each stage agree (convergence), offer complementary information on the same issue (complementarity) or appear to contradict each other (discrepancy or dissonance).^29^

Triangulation of findings will be conducted based on Farmer *et al*’s triangulation protocol.^30^ This involves identifying themes from each stage of the study and then sorting them into similar categories. The themes will be ‘convergence coded’ to identify where there is agreement, silence and dissonance in terms of data from the different stages of the study.^30^ This technique for triangulation is the only one to include silence or where a theme or finding arises from one data set and not another. Silence might be expected because of the strengths of each method to examine different aspects of delegation, but surprise silences might also arise that help to increase understanding.^29^ The format of the framework, for example, paper document or online tool, will be guided by data from stages 1 and 2a and consultation with the Clinical Advisory Group and PPIE group. This will then be formally tested in postdoctoral research. It is anticipated that stage 3 will be completed by June 2024.

### Patient and Public Involvement and Engagement

Patients and/or the public were/will be involved in the design, or conduct, or reporting, or dissemination plans of this research. The researchers have worked with a group of seven patients to develop this mixed-methods research study protocol. They all have experience of treatment by physiotherapists and/or physiotherapy assistants/support workers for an MSK condition.

The PPIE members have reviewed the patient participant information leaflets and consent forms associated with this protocol. The researchers will continue working with the PPIE group to:

- Produce/amend the appropriate materials to inform participants and the public about the study.
- Analyse/interpret the data from the patient interviews and the DCE.
- Design the best practice framework of delegation.
- Produce the materials for sharing the results publicly and decide where to share the results.

### Clinical Advisory Group

A group of clinicians consisting of four physiotherapists, three physiotherapy assistants/support workers, one physiotherapy manager, one clinical lead and a professional advisor from the CSP have helped shape the research plans. The group will continue providing support to this study including supporting its delivery, interpretation of results and dissemination.

### Ethics and dissemination

Written informed consent will be obtained prior to the participants undergoing any activities that are specifically for the purposes of the study. The study requires ethical approval for stages 1, 2a and 2b. Approvals have been received from the South West-Frenchay Research Ethics Committee (REC) (17 December 2021 IRAS ID: 297095, REC reference 21/SW/0158 and 29 December 2022, amendment number AM02 SA01).

Publications reporting on each stage of the study will be prepared for peer-reviewed open-access journals. Additionally, abstracts will be submitted for presentation at local, national and international conferences such as Physiotherapy UK, Health Services Research UK and the World Confederation for Physical Therapy Congress. The results will be shared with the appropriate professional groups and networks within the CSP and at participating sites through in-service training. A webinar will be prepared for physiotherapists and physiotherapy assistants/support workers and will be shared via the CSP’s website and on social media. Furthermore, the results will be presented at local dissemination events that will be organised in each participating Trust involving all key stakeholders. Finally, a plain English summary of the results will be shared with participants and the public via social media, selected magazines and/or newspapers as well as MSK patient groups via Versus Arthritis or other charities.

## Supplementary Material

Reviewer comments

Author's manuscript

## References

[1] Versus arthritis 2019 the state of musculoskeletal health. 2019. Available: www.versusarthritis.org/media/14594/state-of-musculoskeletal-health-2019.pdf [Accessed 18 Jun 2021].

[2] The chartered society of physiotherapy (CSP) 2013 physiotherapy works: musculoskeletal disorders. Available: www.csp.org.uk/system/files/csp_physiotherapy_works_msk_june_2013.pdf [Accessed 18 Jun 2021].

[3] Sarigiovannis P , Cropper S . An audit of the utilization of physiotherapy assistants in the musculoskeletal outpatients setting within a primary care physiotherapy service. Musculoskeletal Care 2018;16:405–8. 10.1002/msc.1238 Available: 10.1002/msc.1238 29532587

[4] The chartered society of physiotherapy (CSP) 2017 information paper PD126 supervision, accountability & delegation. Available: www.csp.org.uk/system/files/supervision_accountability_delegation_final.pdf [Accessed 18 Jun 2021].

[5] Sarigiovannis P , Jowett S , Saunders B , et al . Delegation by allied health professionals to allied health assistants: a mixed methods systematic review. Physiotherapy 2021;112:16–30. 10.1016/j.physio.2020.10.002 Available: 10.1016/j.physio.2020.10.002 34020200

[6] The NHS long term plan 2019 NHS england. 2019. Available: www.longtermplan.nhs.uk/wp-content/uploads/2019/08/nhs-long-term-plan-version-1.2.pdf [Accessed 18 Jun 2021].

[7] Buchan J , Dal Poz MR . Skill mix in the health care workforce: reviewing the evidence. In: Bulletin of the World Health Organisation 80. 2002: 575–80. Available: https://www.who.int/hrh/documents/skill_mix.pdf [accessed 18 Jun 2021]. PMC2567564 12163922

[8] Nancarrow SA , Borthwick AM . Dynamic professional boundaries in the healthcare workforce. Sociol Health Illn 2005;27:897–919. 10.1111/j.1467-9566.2005.00463.x Available: 10.1111/j.1467-9566.2005.00463.x 16313522

[9] Creswell JW , Plano Clark VL . Designing and conducting mixed methods research. International student edition (Third edition). Los Angeles: Sage Publications, 2018.

[10] Rashid M , Hodgson CS , Luig T . Ten tips for conducting focused ethnography in medical education research. Med Educ Online 2019;24:1624133. 10.1080/10872981.2019.1624133 Available: 10.1080/10872981.2019.1624133 31146655 PMC6567138

[11] Knoblauch H . Focused ethnography. In: Forum Qualitative Social Research (FQS) 6. 2005: 44. 10.17169/fqs-6.3.20

[12] Higginbottom GMA , Pillay JJ , Boadu NY . Guidance on performing focused ethnographies with an emphasis on healthcare research. TQR 2013:1–16. 10.46743/2160-3715/2013.1550 Available: 10.46743/2160-3715/2013.1550

[13] Finlay L . “Outing” the researcher: the provenance, process, and practice of reflexivity. Qual Health Res 2002;12:531–45. 10.1177/104973202129120052 Available: 10.1177/104973202129120052 11939252

[14] Saunders B , Sim J , Kingstone T , et al . Saturation in qualitative research: exploring its conceptualization and operationalization. Qual Quant 2018;52:1893–907. 10.1007/s11135-017-0574-8 Available: 10.1007/s11135-017-0574-8 29937585 PMC5993836

[15] Roper JM , Shapira J . Ethnography in nursing research. 2455 Teller Road, Thousand Oaks California 91320 United States: Sage Publications, 2000. 10.4135/9781483328294

[16] Protheroe J , Saunders B , Bartlam B , et al . Matching treatment options for risk sub-groups in musculoskeletal pain: a consensus groups study. BMC Musculoskelet Disord 2019;20:271. 10.1186/s12891-019-2587-z Available: 10.1186/s12891-019-2587-z 31153364 PMC6545223

[17] McMillan SS , Kelly F , Sav A , et al . Using the nominal group technique: how to analyse across multiple groups. Health Serv Outcomes Res Method 2014;14:92–108. 10.1007/s10742-014-0121-1 Available: 10.1007/s10742-014-0121-1 PMC4282516 25281284

[18] List D . The consensus group technique in social research. Field Methods 2001;13:277–90. 10.1177/1525822X0101300304 Available: 10.1177/1525822X0101300304

[19] Williamson PR , Altman DG , Blazeby JM , et al . Developing core outcome sets for clinical trials: issues to consider. Trials 2012;13:132. 10.1186/1745-6215-13-132 Available: 10.1186/1745-6215-13-132 22867278 PMC3472231

[20] Van Breda AD . Steps to analysing multiple-group NGT data. Soc Work Pract Res 2005;17:1–14.

[21] Ryan M , Bate A , Eastmond CJ , et al . Use of discrete choice experiments to elicit preferences. Qual Health Care 2001;10 Suppl 1(Suppl 1):i55–60. 10.1136/qhc.0100055 11533440 PMC1765744

[22] de Bekker-Grob EW , Donkers B , Jonker MF , et al . Sample size requirements for discrete-choice experiments in healthcare: a practical guide. Patient 2015;8:373–84. 10.1007/s40271-015-0118-z Available: 10.1007/s40271-015-0118-z 25726010 PMC4575371

[23] Coast J , Al-Janabi H , Sutton EJ , et al . Using qualitative methods for attribute development for discrete choice experiments: issues and recommendations. Health Econ 2012;21:730–41. 10.1002/hec.1739 Available: 10.1002/hec.1739 21557381

[24] Soekhai V , de Bekker-Grob EW , Ellis AR , et al . Discrete choice experiments in health economics: past, present and future. Pharmacoeconomics 2019;37:201–26. 10.1007/s40273-018-0734-2 Available: 10.1007/s40273-018-0734-2 30392040 PMC6386055

[25] de Bekker-Grob EW , Swait JD , Kassahun HT , et al . Are healthcare choices predictable? the impact of discrete choice experiment designs and models. Value Health 2019;22:1050–62.:S1098-3015(19)32147-3. 10.1016/j.jval.2019.04.1924 Available: 10.1016/j.jval.2019.04.1924 31511182

[26] Mentzakis E , Ryan M , McNamee P . Using discrete choice experiments to value informal care tasks: exploring preference heterogeneity. Health Econ 2011;20:930–44. 10.1002/hec.1656 Available: 10.1002/hec.1656 20799343

[27] Lancsar E , Louviere J . Conducting discrete choice experiments to inform healthcare decision making: a user’s guide. Pharmacoeconomics 2008;26:661–77. 10.2165/00019053-200826080-00004 Available: 10.2165/00019053-200826080-00004 18620460

[28] Hole AR . Modelling heterogeneity in patients’ preferences for the attributes of a general practitioner appointment. J Health Econ 2008;27:1078–94. 10.1016/j.jhealeco.2007.11.006 Available: 10.1016/j.jhealeco.2007.11.006 18179837

[29] O’Cathain A , Murphy E , Nicholl J . Three techniques for integrating data in mixed methods studies. BMJ 2010;341:bmj.c4587. 10.1136/bmj.c4587 Available: 10.1136/bmj.c4587 20851841

[30] Farmer T , Robinson K , Elliott SJ , et al . Developing and implementing a triangulation protocol for qualitative health research. Qual Health Res 2006;16:377–94. 10.1177/1049732305285708 Available: 10.1177/1049732305285708 16449687

