# Supplementary material for: Developing a best practice framework for musculoskeletal outpatient physiotherapy delegation: the MOPeD mixed-methods research study protocol
Source: BMJ Open. 2023 Mar 17;13(3):e072989. doi: 10.1136/bmjopen-2023-072989 (PMC10030620; doi:10.1136/bmjopen-2023-072989)
Supplement: Supplementary data [file bmjopen-2023-072989supp001.pdf]

NHS Midlands Partnership NHS Foundation Trust A Keele University Teaching Trust

MOPeD STUDY

Keele UNIVERSITY

Musculoskeletal Outpatient Physiotherapy Delegation (MOPeD) (Stage 1, Focused Ethnography)

Topic Guide: Physiotherapists

1. Introduction

Check that participant has read and understood the Participant Information Leaflet (patient version)
Explain arrangements for: consent, recording, anonymity, expenses where appropriate etc.

2. Training on delegating clinical tasks to physiotherapy assistants (where possible invite participants to expand on their responses)

1) Can you tell me about your experience as a physiotherapist i.e. how many years have you worked as a physio?
2) How many years have you worked in the MSK setting?
3) Have you had any formal or informal training in relation to delegating clinical tasks to physiotherapy assistants? Probe for types of training e.g. formal/informal, when etc.

3. Experience and acceptability of delegating tasks to physiotherapy assistants (where possible invite participants to expand on their responses)

4) Do you always delegate clinical tasks to physiotherapy assistants?
5) Is this a personal choice or do you follow agreed treatment pathways?
6) Are you aware of any professional guidance about delegation? If yes, can you name it?
7) How do you feel about delegating part of your clinical treatment? If yes/no, can you explain why?
8) Are there any parts of the treatment plan that you do not delegate? Why?
9) Can you talk about a complete treatment where delegation worked really well? Why did it work well?
10) Can you recall a case where delegation did not work so well? Why do you think this happened?
11) How do you feel about delegating clinical tasks to physiotherapy assistants?

4. Close of discussion

a. Summary of discussion: any additional remarks?
b. Check consent is still in place.
c. Check if participant would like to receive a summary of the interview findings.

FUNDED BY NIHR | National Institute for Health Research
