# Supplementary material for: Developing a best practice framework for musculoskeletal outpatient physiotherapy delegation: the MOPeD mixed-methods research study protocol
Source: BMJ Open. 2023 Mar 17;13(3):e072989. doi: 10.1136/bmjopen-2023-072989 (PMC10030620; doi:10.1136/bmjopen-2023-072989)
Supplement: Author's manuscript [file bmjopen-2023-072989.draft_revisions.pdf]

BMJ Open is committed to open peer review. As part of this commitment we make the peer review history of every article we publish publicly available.

When an article is published we post the peer reviewers' comments and the authors' responses online. We also post the versions of the paper that were used during peer review. These are the versions that the peer review comments apply to.

The versions of the paper that follow are the versions that were submitted during the peer review process. They are not the versions of record or the final published versions. They should not be cited or distributed as the published version of this manuscript.

BMJ Open is an open access journal and the full, final, typeset and author-corrected version of record of the manuscript is available on our site with no access controls, subscription charges or pay-per-view fees (http://bmjopen.bmj.com).

If you have any questions on BMJ Open's open peer review process please email info.bmjopen@bmj.com

BMJ Open

Developing a best-practice framework for Musculoskeletal Outpatient Physiotherapy Delegation: the MOPeD mixed methods research study protocol

Journal:	*BMJ Open*
Manuscript ID	bmjopen-2023-072989
Article Type:	Protocol
Date Submitted by the Author:	20-Feb-2023
Complete List of Authors:	Sarigiovannis, Panos; Keele University, Primary Care Centre Versus Arthritis, School of Medicine; Midlands Partnership NHS Foundation Trust, North Integrated Musculoskeletal Service<br>Foster, Nadine; The University of Queensland and Metro North Health, STARS Education and Research Alliance, Surgical Treatment and Rehabilitation Service (STARS); Keele University, Primary Care Centre Versus Arthritis, School of Medicine<br>Jowett, Sue; University of Birmingham, Health Economics Unit, Institute of Applied Health Research, Public Health Building<br>Saunders, Benjamin; Keele University, Primary Care Centre Versus Arthritis, School of Medicine
Keywords:	Adult orthopaedics < ORTHOPAEDIC & TRAUMA SURGERY, Musculoskeletal disorders < ORTHOPAEDIC & TRAUMA SURGERY, Health & safety < HEALTH SERVICES ADMINISTRATION & MANAGEMENT

SCHOLARONE™
Manuscripts

TITLE

Developing a best-practice framework for Musculoskeletal Outpatient Physiotherapy Delegation: the MOPeD mixed methods research study protocol

Author(s):

Panos Sarigiovannis, Clinical Doctoral Research Fellow[1,2], Musculoskeletal Clinical Specialist Physiotherapist[2]

Nadine E Foster, Professor and Director, STARS Education and Research Alliance[3], Visiting Professor of Musculoskeletal Health[1]

Sue Jowett, Professor of Health Economics[4]

Benjamin Saunders, Lecturer in Applied Health Research and Deputy Director of the Centre for Musculoskeletal Health Research[1]

Institute(s):

1. Primary Care Centre Versus Arthritis, School of Medicine, Keele University, Staffordshire, ST5 5BG, United Kingdom.
2. Midlands Partnership NHS Foundation Trust, Newcastle under Lyme, Staffordshire, ST5 2BQ, United Kingdom.
3. STARS Education and Research Alliance, Surgical Treatment and Rehabilitation Service (STARS), The University of Queensland and Metro North Health, Herston, Brisbane, Australia.
4. Health Economics Unit, Institute of Applied Health Research, Public Health Building, University of Birmingham, Edgbaston, Birmingham, B15 2TT, United Kingdom.

Authors' email addresses

P Sarigiovannis: p.sarigiovannis1@keele.ac.uk

NE Foster: n.foster@uq.edu.au

S Jowett: s.jowett@bham.ac.uk

B Saunders: b.saunders@keele.ac.uk

Corresponding author:

Sarigiovannis P

ORCID: https://orcid.org/0000-0001-7960-1500

For peer review only - http://bmjopen.bmj.com/site/about/guidelines.xhtml

Address: Primary Care Centre Versus Arthritis, School of Medicine, Keele University, Staffordshire, ST5 5BG, United Kingdom.

Email: p.sarigiovannis1@keele.ac.uk

Twitter: @sarigiovannis1

Word count: 3919

For peer review only

For peer review only - http://bmjopen.bmj.com/site/about/guidelines.xhtml

ABSTRACT

Introduction: Physiotherapy assistants/support workers are an important part of the physiotherapy workforce in the UK. Many of them work in NHS physiotherapy outpatient services treating patients with musculoskeletal (MSK) conditions. In many services they take responsibility, under professional supervision, for types of clinical work traditionally undertaken by physiotherapists such as leading exercise classes and treating individual patients. Nevertheless, their role(s) are relatively undefined and as such there is considerable variation in the duties and tasks they undertake. This study aims to design a framework of 'best practice' in delegation to guide the work of clinicians in NHS physiotherapy MSK services and facilitate standardisation of practice to ensure that patients receive safe and effective treatment by the most appropriate person.

Methods and analysis: This mixed methods study will be conducted in four stages. In stage 1, a focused ethnography in two MSK outpatient physiotherapy services will explore how the current use of delegation is informed by the culture within the clinical setting as well as views, attitudes about, and experiences of, delegation among clinicians, managers and patients. In stage 2a, Nominal Group Technique will be used with three separate groups (physiotherapists/physiotherapy assistants/support workers, managers) to reach consensus about what components should be included in a best practice framework of delegation. In stage 2b, a discrete choice experiment will elicit patients' preferences between care from physiotherapists and physiotherapy assistants/support workers within MSK physiotherapy services. In the final stage, the results of all previous stages will be triangulated to inform the development of a best practice delegation framework for future testing and use within NHS MSK outpatient physiotherapy services.

Ethics and dissemination: Ethical approval has been granted by the South West - Frenchay Research Ethics Committee. The findings will be disseminated in peer-reviewed journals, conference presentations, the lay press and social media.

For peer review only - http://bmjopen.bmj.com/site/about/guidelines.xhtml

Key words: Skill mix, personnel delegation, physiotherapy support workers, mixed methods, protocol

For peer review only

For peer review only - http://bmjopen.bmj.com/site/about/guidelines.xhtml

ARTICLE SUMMARY

Strengths and limitations of this study

- A mixed methods study which includes qualitative and quantitative data and multiple research approaches will enable a more comprehensive understanding of the issues that affect delegation in the MSK physiotherapy setting
- The design of a framework of best practice in delegation to guide the work of physiotherapy assistants/support workers in the MSK physiotherapy setting, underpinned by patients' preferences and clinicians' consensus, may facilitate successful implementation in clinical practice
- The focused ethnographic study is being conducted across two NHS Trusts and the Discrete Choice Experiment across one NHS Trust therefore, the results may not be representative of the NHS more broadly
- Patients and clinicians have been involved in designing the research and they will continue to be involved in all stages of the study

For peer review only

For peer review only - http://bmjopen.bmj.com/site/about/guidelines.xhtml

1 INTRODUCTION

Musculoskeletal (MSK) conditions such as low back pain and osteoarthritis affect one in four people globally, are increasingly common with age, are the leading cause of pain and disability in the UK and the second leading cause of sickness absence from work [1]. Patients with MSK conditions are the largest patient population group treated by physiotherapists [2]. Patients are assessed by physiotherapists and if they need follow-up treatments, they are usually treated by either a physiotherapist or a physiotherapy support worker. Physiotherapy support workers are non-registered staff who work alongside physiotherapists to provide delegated interventions and responsibilities. They may also be known as physiotherapy assistants, rehabilitation assistants, technical instructors or physiotherapy technicians. There are approximately 9,000 physiotherapy assistants/support workers in the UK, forming 15% of the total physiotherapy workforce and a large proportion of them work in the NHS. In many services they take responsibility, under professional supervision, for certain types of clinical work traditionally undertaken by qualified physiotherapists, such as leading exercise classes and treating individual patients. However, their role(s) are relatively undefined and as such, there is considerable variation in the duties and tasks that they undertake [3]. National guidance from the Chartered Society of Physiotherapy (CSP)[1] about delegation of tasks to physiotherapy assistants/support workers largely leave decision-making to the individual physiotherapist, their judgement of the task and their assessment of the competence of the physiotherapy assistant/support worker [4]. As a result, in some physiotherapy services, physiotherapy assistants/support workers have a predominantly clinical role whereas in others they fulfil primarily an administrative role such as data inputting and booking appointments. This latter situation leads to physiotherapy assistants/support workers not being able to utilise their clinical skills, experiencing job dissatisfaction, as well as to unjustified variation in care and clinical services provided to

[1] CSP is the professional, educational and trade union body for the UK's chartered physiotherapists, physiotherapy students and support workers.

For peer review only - http://bmjopen.bmj.com/site/about/guidelines.xhtml

patients [3]. Results from a recent systematic review which explored the clinical and cost-effectiveness and perceptions of delegation by Allied Health Professionals to Allied Health Assistants internationally [5], highlighted that delegation is not standardised within physiotherapy and that there are clear knowledge gaps regarding delegation by physiotherapists in current practice. These relate to the clinical and cost-effectiveness of delegation as well as patients' preferences, experiences of and attitudes about delegation. This study aims to design a framework of best practice in delegation to guide the work of physiotherapy assistants/support workers in NHS physiotherapy MSK services and facilitate standardisation of practice to ensure that patients receive safe and effective treatment by the most appropriate person.

2 RATIONALE

The NHS Long Term Plan includes a commitment to narrow health inequalities and address unwarranted variation in care [6]. A framework of best practice for delegation within the MSK setting could facilitate standardisation of delegation in physiotherapy and therefore, minimise unwarranted variation in the provision of physiotherapy services. Appropriate use of physiotherapy assistants/support workers could reduce healthcare costs either directly or indirectly since it could release capacity for physiotherapists to treat patient cases that are more complex or to be the first point of contact for some patients, in place of a general practitioner (GP) or a consultant doctor. Most importantly, patients would see the right staff with the right skills at the right time, which could potentially optimise clinical outcomes and patient satisfaction. Since staffing costs usually account for between 60% and 80% of operating costs in healthcare services, determining the 'right' combination of staff with the right skills is a critical component of successful and efficient healthcare delivery [7, 8]. The findings of this research will guide workforce planning in MSK physiotherapy services and identify future training needs. Finally, the best practice delegation framework will underpin a

For peer review only - http://bmjopen.bmj.com/site/about/guidelines.xhtml

future research study which will assess the clinical and cost-effectiveness of delegation using this framework in clinical settings.

3 THEORETICAL FRAMEWORK

An exploratory sequential mixed methods design will be used. This is characterised by an initial qualitative phase of data collection and analysis followed by a quantitative phase. It also includes a final phase of integration or linking the data from the two separate strands of data. The conceptual framework in the exploratory design will be inductively developed in the initial phase of the study where qualitative data results may lead to a theoretical model [9]. One of the strengths of the exploratory sequential design is that the researcher can produce a new instrument or a framework as one of the potential products of the research process [9].

4 RESEARCH QUESTION/AIM(S)

The overall research question is:

> 'What should a 'best practice' framework of delegation incorporate and how can this be operationalised to guide utilisation of physiotherapy assistants/support workers in NHS MSK outpatient physiotherapy services?'

4.1 Objectives

The specific objectives are to:

1. Explore how the use of delegation is informed by the culture within the clinical setting as well as perceptions of, and attitudes about, delegation among physiotherapists, physiotherapy assistants/support workers, physiotherapy managers and patients.

For peer review only - http://bmjopen.bmj.com/site/about/guidelines.xhtml

2. Reach consensus between physiotherapists, physiotherapy assistants/support workers and physiotherapy managers about what constitutes “best practice” and what components should be included in a best practice framework of delegation in NHS MSK outpatient physiotherapy services.

3. Explore patients’ preferences in relation to delegation in NHS MSK outpatient physiotherapy services and estimate specific trade-offs patients are willing to make in treatment choices when they are treated by physiotherapy assistants/support workers.

4. Develop a best practice delegation framework, which can be tested in future research and used within NHS MSK outpatient physiotherapy services.

An outline of the MOPeD study is shown in figure 1.

[Figure 1 to be inserted here]

For peer review only

For peer review only - http://bmjopen.bmj.com/site/about/guidelines.xhtml

5 METHODS AND ANALYSIS

5.1 Eligibility criteria

The eligibility criteria for each stage of the MOPeD study are shown in table 1.

[Table 1 to be inserted here]

Table 1: Eligibility Criteria for each stage

MOPeD study eligibility criteria	
**Stage 1: Focused ethnography**	1. Adult patients (18 years old or older) with an MSK condition who attend physiotherapy appointments at participating clinics during the period of the study.<br>2. Physiotherapists, physiotherapy assistants/support workers and physiotherapy managers working in participating MSK outpatient physiotherapy services.
**Stage 2a: Consensus study**	1. Experienced physiotherapists (Agenda for Change[2] band 6 or higher) working in the UK NHS in the treatment of MSK conditions.<br>2. Physiotherapy assistants/support workers working in the UK NHS, treating patients with MSK conditions.<br>3. Physiotherapy service managers working in the UK NHS outpatient MSK physiotherapy Services.
**Stage 2b: Discrete choice experiment**	Adult patients (18 years old or older) who have been offered a follow-up physiotherapy appointment and/or completed a course of physiotherapy treatment for a MSK condition in one of the MSK outpatient physiotherapy clinics within the participating NHS Trust.

5.2 Stage 1

5.2.1 Design: A focused ethnographic study

A focused ethnography will be conducted in two MSK outpatient physiotherapy services.

Focused ethnography explores a specific issue, situation or problem within a specific context

[2] Physiotherapists working in the NHS are employed under the Agenda for Change (AfC) grading and pay system where higher bandings are associated with higher qualifications and pay. Newly qualified physiotherapists work in Band 5 positions, while physiotherapists who have completed their junior rotations are in band 6 positions.

For peer review only - http://bmjopen.bmj.com/site/about/guidelines.xhtml

[10, 11]. It is very suitable for healthcare research as it provides an efficient way to capture in-depth data on a specific topic of importance to individual clinicians or clinical specialities, and to determine ways to improve care and care processes [12]. This study has a clear focus on delegation, in a specific clinical setting and the research team has prior experience of using and organising clinical delegation. Therefore, focused ethnography will enable the researchers to explore how the use of delegation is informed by the culture of two different clinical settings, which will allow for the development of an in-depth and comparative understanding about how delegation is currently being used, and the factors that influence delegation to physiotherapy assistants/support workers at an individual, collective, and broader organisation level. It will also allow the researchers to better understand attitudes about, and experiences of, delegation among physiotherapists, physiotherapy assistants/support workers, physiotherapy managers and patients.

5.2.2 Data collection

Stage 1/Focused ethnography

A purposive sampling design has been selected as two physiotherapy services that are known to use delegation successfully (positive deviance approach) will be observed. These were chosen in collaboration with the appropriate professional advisor from the CSP who currently leads the support workers' workstream, taking into consideration different indicators such as continuous use of physiotherapy assistants/support workers in treating patients with MSK conditions and dissemination of clinical practice. Within stage 1, criterion sampling will be used as the purposeful sampling design for the selection of patients who will be invited to participate in the observations and/or interviews as only patients who have been treated by a physiotherapist and a physiotherapy assistant/support worker will be invited.

The researcher will use participant observation in two clinical sites focusing on current delegation practice, semi-structured interviews with physiotherapists, physiotherapy

For peer review only - http://bmjopen.bmj.com/site/about/guidelines.xhtml

assistants/support workers, managers and patients, as well as a review of physiotherapy treatment records and clinical site records such as policies, job descriptions and delegation training records to gain a rich and comprehensive understanding of how delegation works in the observed setting. Whenever appropriate, similarities and differences across the different types of data will be explored. Field observation will involve observing a range of activities including clinical sessions (one to one or groups) that physiotherapy assistants/support workers deliver independently or participate in, supervision sessions, training sessions and team meetings. Field notes will include information about observed events but also the researcher's personal reflections and interpretation of events [13].

All physiotherapy assistants/support workers who work in the observed clinical setting, the physiotherapists who delegate clinical tasks to them, the operational manager(s)/team leader(s) and patients will be invited to participate in the interviews. Up to 12 patients and 12 clinicians (physiotherapists, physiotherapy assistants/support workers, manager/s) will be interviewed at each site (i.e. up to 24 interviews in total per site). A final decision on sample size will be made once data collection and analyses are ongoing, based on data saturation [14].

The interview schedule will be developed based on the evidence from a systematic review conducted by Sarigiovannis et al [5]. The interviews will give the researcher the opportunity to ask physiotherapists, physiotherapy assistants/support workers, managers, and patients for elaborations about specific topics, explanations of observed events and clarification of ambiguities [15]. Questions will cover topics such as how the tasks were delegated, how patients feel about the delegation of clinical tasks etc. The questions will be reviewed by the study's Patient and Public Involvement and Engagement (PPIE) and Clinical Advisory Groups to ensure acceptability of the questions and ease of understanding. The examination of relevant documents such as patients' physiotherapy treatment records and relevant policies will help validate the data from observations and interviews.

For peer review only - http://bmjopen.bmj.com/site/about/guidelines.xhtml

5.2.3 Data analysis

Data analysis will be based on the approach described by Roper and Shapira [15]. Analytical steps include coding field notes and interviews, sorting to identify patterns for descriptive labels, identifying outliers or cases that do not "fit" with the rest of the findings, generalising in relation to existing concepts and theories as well as the ideas and insights the researcher has about the data, including their reflective field notes [15]. The analytical steps will be focused on answering specific problem-orientated research questions and the creation of concrete recommendations. The qualitative data analysis software QSR NVivo will be used to facilitate analysis.

5.3 Stage 2a

5.3.1 Design: A Nominal Group Technique (NGT) approach

A consensus study will be carried out, using Nominal Group Technique (NGT) a systematic approach to building consensus using a structured set of stages. A convenience sample of physiotherapists, physiotherapy assistants/support workers and physiotherapy managers who work in NHS MSK Physiotherapy Services will be recruited through the CSP's professional networks and through social media (Facebook, Twitter and LinkedIn) accounts of the authors and their networks. Three separate role-specific NGT working groups will be convened, with; 1) physiotherapists; 2) managers; and 3) physiotherapy assistants/support workers with the aim to reach agreement about what a 'best practice' delegation framework should include. Each participant group will take part in a single meeting lasting approximately two hours. Two facilitators will lead each meeting. They will use a 'pre-elicitation technique' to inform the participants' decision-making [16]. Specifically, prior to the meeting participants will receive a summary of existing evidence based on the findings from a systematic review conducted by Sarigiovannis et al [5]. The participants will initially be asked to silently generate ideas about what should be included in a delegation framework for use within the

For peer review only

MSK physiotherapy setting, which will then be shared with the group one by one. Each shared item will then be individually rated by all participants. Following this, the results of these ratings will be discussed within the group, and then they will be re-rated individually by participants, who will be given the opportunity to amend their scores in light of group discussions.

The rating process will be explained step-by-step and participants will be given enough time for voting [17]. Each item will be rated on a 7-point Likert scale by individual participants and a mean rating calculated. The threshold for consensus will be set at ≥70% i.e. ≥ 4.9 on the 7-point Likert scale across the group [18,19]. The data from the 3 groups will be combined into one complete sample to identify the most highly prioritised items [20]. This includes calculating the mean scores for the raw data, the mean scores for the themes and the frequency of themes (i.e. how many times a theme appeared in the top five and how often an idea was raised and coded under the same theme). All items will be categorised using a modified thematic analysis approach which will include generating initial themes from the collated data and refining themes. The items will be rated using an online voting platform called Mentimeter (https://www.mentimeter.com/) which participants will access on their smartphones or laptops. The issues discussed during the meeting will not include any topics that might be sensitive, embarrassing or upsetting. The facilitators will keep notes during the consensus meetings. A list of all items that reached consensus categorised into themes will be the final output from the consensus study.

5.4 Stage 2b

5.4.1 Design: A Discrete Choice Experiment (DCE)

Discrete choice experiments (DCEs) are an attribute-based survey method for measuring benefits (utility). Within healthcare, the technique is applied to address a wide range of issues in the delivery of healthcare including measuring and valuing attributes of a

For peer review only - http://bmjopen.bmj.com/site/about/guidelines.xhtml

healthcare service and identifying the factors that influence choices and decisions of patients, the public and healthcare professionals [21]. They are based on the assumption that a service can be described by its characteristics or attributes, and the extent to which an individual values the service depends on the levels of these characteristics [21]. In a DCE, respondents are asked to choose between two or more choice sets.

A DCE will be designed based on the data from the ethnography as well as further input from the study's Clinical Advisory Group and PPIE group. The DCE will be conducted to elicit patients' preferences about the use of delegation to physiotherapy assistants/support workers within MSK physiotherapy services. Convenience sampling will be used during this stage to invite patients who have been offered a follow up physiotherapy appointment and/or completed a course of physiotherapy treatment programme in one of the MSK outpatient physiotherapy services within the participating NHS Trust. Patients will be invited by the treating clinicians when patients attend their follow up physiotherapy appointment/are discharged. The minimum sample size needed for the DCE depends on the specific hypotheses to be tested [22]. Therefore, the power of the DCE will be calculated when the questionnaire is finalised. It is expected that the number of participants will not exceed 500. This number is feasible since approximately 1500 patients are discharged per month within the Trust.

5.4.2 Data collection

Development of the attributes and levels will be undertaken using the appropriate findings from a systematic review on delegation by Allied Health Professionals (AHPs) to support workers [5]; the ethnographic data from stage 1 of the study; and further input from the study's PPIE group [23]. A recent systematic review of DCEs in healthcare reported that most studies included four to nine attributes, and among them four to five was the modal category [24]. The intention is to select between five to seven attributes. The Ngene design software will be used to create the choice sets. In addition to the DCE, the survey will include items to elicit sociodemographic information such as age, gender, and employment status as

For peer review only

For peer review only - http://bmjopen.bmj.com/site/about/guidelines.xhtml

well as experience of being treated by a physiotherapy assistant/support worker. These seem to play an important role in predicting healthcare choices [25].

The DCE survey will be offered in an online format, completed in physiotherapy clinics using tablet devices. The online version will be saved on a secure server. The understanding of the attributes and levels will be pretested among members of the PPIE group. The survey will then be piloted with a small sample of patients with MSK conditions consulting physiotherapy services. This will include testing respondent understanding of the different choices offered, generation and testing of appropriateness and understanding of attributes/levels, task complexity, length, timing and likely response rates [26, 27].

5.4.3 Data analysis

Data analysis will be completed using the STATA software. Although preference heterogeneity has long been accounted for in the analysis of DCEs by interacting design attributes with socio-demographic characteristics, evidence suggests that this approach only partially accounts for the differences in preference embodied in the data [28]. Research has shown that DCE models that take into account both preference heterogeneity and differences in the error variance of choices (scale heterogeneity) are better to predict choices mimicking real-world decisions [23]. Therefore, selecting the appropriate model for data analysis is important. The choice of the model will be finalised following discussions with DCE experts. The results will help the researchers understand what influences patients' preferences by estimating the value patients place on key attributes and associated attribute levels. The researchers will estimate specific trade-offs that patients who are treated in NHS MSK outpatient physiotherapy services are willing to make in treatment choices, specifically in relation to being treated by physiotherapy assistants/support workers.

For peer review only - http://bmjopen.bmj.com/site/about/guidelines.xhtml

5.5 Stage 3

5.5.1 Design and analysis: Development of a best practice framework of delegation

The results from stages 1, 2a and 2b will be triangulated to inform the design of a best-practice delegation framework for NHS MSK outpatient physiotherapy services. Triangulation will involve listing the findings from each stage of the study and consider where findings from each stage agree (convergence), offer complementary information on the same issue (complementarity), or appear to contradict each other (discrepancy or dissonance) [29].

Triangulation of findings will be conducted based on Farmer et al's triangulation protocol [30]. This involves identifying themes from each stage of the study and then sorting them into similar categories. The themes will be "convergence coded" to identify where there is agreement, silence and dissonance in terms of data from the different stages of the study [30]. This technique for triangulation is the only one to include silence or where a theme or finding arises from one data set and not another. Silence might be expected because of the strengths of each method to examine different aspects of delegation, but surprise silences might also arise that help to increase understanding [29]. The format of the framework e.g. paper document or online tool, will be guided by data from stages 1 and 2a and consultation with the Clinical Advisory and PPIE groups. This will then be formally tested in post-doctoral research.

5.6 Patient and Public Involvement and Engagement

The researchers have worked with a group of seven patients to develop this mixed methods research study protocol. They all have experience of treatment by physiotherapists and/or physiotherapy assistants/support workers for a MSK condition.

For peer review only - http://bmjopen.bmj.com/site/about/guidelines.xhtml

The PPIE members have reviewed the patient participant information leaflets and consent forms associated with this protocol. The researchers will continue working with the PPIE group to:

- Produce/amend the appropriate materials to inform participants and the public about the study
- Analyse/interpret the data from the patient interviews and the DCE
- Design the best practice framework of delegation
- Produce the materials for sharing the results publicly and decide where to share the results

5.7 Clinical Advisory Group

A group of clinicians consisting of four physiotherapists, three physiotherapy assistants/support workers, one physiotherapy manager, one clinical lead, and a Professional Advisor from the CSP have helped shape the research plans. The group will continue providing support to this study including supporting its delivery, interpretation of results and dissemination.

6 ETHICS AND DISSEMINATION

Informed consent will be obtained prior to the participants undergoing any activities that are specifically for the purposes of the study. The study requires ethical approval for stages 1, 2a and 2b. Approvals have been received from the South West - Frenchay Research Ethics Committee (17 December 2021 IRAS ID: 297095, REC Reference 21/SW/0158 & 29 December 2022, Amendment number AM02 SA01).

Publications reporting on each stage of the study will be prepared for peer-reviewed open access journals. Additionally, abstracts will be submitted for presentation at local, national and international conferences such as Physiotherapy UK, Health Services Research UK and

For peer review only - http://bmjopen.bmj.com/site/about/guidelines.xhtml

the World Confederation for Physical Therapy Congress. The results will be shared with the appropriate professional groups and networks within the CSP and at participating sites through in-service-training. A webinar will be prepared for physiotherapists and physiotherapy assistants/support workers and will be shared via the CSP's website and on social media. Furthermore, the results will be presented at local dissemination events that will be organised in each participating Trust involving all key stakeholders. Finally, a plain English summary of the results will be shared with participants and the public via social media, selected magazines and/or newspapers as well as MSK patient groups via Versus Arthritis or other charities.

For peer review only

For peer review only - http://bmjopen.bmj.com/site/about/guidelines.xhtml

Author Contributions

PS wrote the paper and conceived the study with the support of NEF, SJ and BS. All authors have read, edited and approved the final version.

Funding

Panos Sarigiovannis was funded by National Institute for Health and Care Research awards (HEE/NIHR ICA Programme Clinical Doctoral Research Fellowship NIHR301550) for this research project.

Disclaimer

The views expressed in this publication are those of the author(s) and not necessarily those of the NIHR, NHS or the UK Department of Health and Social Care.

Competing interests

None declared.

Patient and public involvement

Patients and/or the public were/will be involved in the design, or conduct, or reporting, or dissemination plans of this research. Refer to the appropriate section for further details.

Data availability statement

No additional data available.

For peer review only

For peer review only - http://bmjopen.bmj.com/site/about/guidelines.xhtml

REFERENCES

[1] Versus Arthritis 2019 The State of Musculoskeletal Health. Arthritis and other Musculoskeletal conditions in number https://www.versusarthritis.org/media/14594/state-of-musculoskeletal-health-2019.pdf [Accessed on 18/06/2021]

[2] The Chartered Society of Physiotherapy (CSP) 2013 Physiotherapy Works: Musculoskeletal Disorders, https://www.csp.org.uk/system/files/csp_physiotherapy_works_msk_june_2013.pdf [Accessed on 18/06/2021]

[3] Sarigiovannis P; Cropper S 2018 An audit of the utilization of physiotherapy assistants in the musculoskeletal outpatients setting within a primary care physiotherapy service. Musculoskeletal Care, 16(3):405–408 https://doi.org/10.1002/msc.1238

[4] The Chartered Society of Physiotherapy (CSP) 2017 Information Paper PD126 Supervision, Accountability & Delegation, https://www.csp.org.uk/system/files/supervision_accountability_delegation_final.pdf [Accessed on 18/06/2021]

[5] Sarigiovannis P, Jowett S, Saunders B, Corp N, Bishop A 2021 Delegation by Allied Health Professionals to Allied Health Assistants: a mixed methods systematic review. Physiotherapy, 112:16-30. https://doi.org/10.1016/j.physio.2020.10.002

[6] NHS England 2019 The NHS Long Term Plan. https://www.longtermplan.nhs.uk/wp-content/uploads/2019/08/nhs-long-term-plan-version-1.2.pdf [Accessed on 18/06/2021]

[7] Buchan J, Dal Poz MR 2002 Skill mix in the health care workforce: reviewing the evidence. Bulletin of the World Health Organisation, 80(7):575-580. https://www.who.int/hrh/documents/skill_mix.pdf [Accessed on 18/06/2021]

[8] Nancarrow SA, Borthwick AM 2005 Dynamic professional boundaries in the healthcare workforce. Sociology of Health & Illness, 27(7):897-919. https://doi.org/10.1111/j.1467-9566.2005.00463.x

[9] Creswell JW, Plano Clark VL 2018 Designing and conducting mixed methods research. International student edition (Third edition). Sage Publications, Los Angeles.

[10] Rashid M, Hodgson CS, Luig T 2019 Ten tips for conducting focused ethnography in medical education research. Medical Education Online, 24(1):1624133 https://doi.org/10.1080/10872981.2019.1624133

[11] Knoblauch H 2005 Focused Ethnography. Forum Qualitative Social Research (FQS); 6 (3):Art. 44 https://doi.org/10.17169/fqs-6.3.20

[12] Higginbottom GMA, Pillay JJ, Boadu NY 2013 Guidance on Performing Focused Ethnographies with an Emphasis on Healthcare Research. The Qualitative Report, 18, Article 17:1-16. https://doi.org/10.46743/2160-3715/2013.1550

[13] Finlay L 2002 "Outing" the Researcher: The Provenance, Process, and Practice of Reflexivity Qualitative Health Research, 12 (4):531-545. https://doi.org/10.1177/104973202129120052

For peer review only - http://bmjopen.bmj.com/site/about/guidelines.xhtml

[14] Saunders B, Sim J, Kingstone T, Baker S, Waterfield J, Bartlam B, Burroughs H, Jinks C 2018 Saturation in qualitative research: exploring its conceptualization and operationalization. Quality & Quantity 52 (4):1893-1907. https://doi.org/10.1007/s11135-017-0574-8

[15] Roper JM, Shapira J 2000 Ethnography in Nursing Research. Sage Publications, Thousand Oaks, California.

[16] Protheroe J, Saunders, B., Bartlam, B, Dunn KM, Cooper V, Campbell P, Hill JC, Tooth S, Mallen CD, Hay EM, Foster NE 2019 Matching treatment options for risk sub-groups in musculoskeletal pain: a consensus groups study. BMC Musculoskeletal Disorders, 20: 271, https://doi.org/10.1186/s12891-019-2587-z

[17] McMillan SS, Kelly F, Sav A, Kendall E, King MA, Whitty JA, Wheeler AJ 2014 Using the Nominal Group Technique: how to analyse across multiple groups. Health Services and Outcomes Research Methodology, 14:92-108. https://doi.org/10.1007/s10742-014-0121-1

[18] List D 2001 The Consensus Group Technique in Social Research. Field Methods, 13 (3): 277–290. https://doi.org/10.1177/1525822X0101300304

[19] PR Williamson DG Altman, JM Blazeby, M Clarke, D Devane, E Gargon, P Tugwell 2012 Developing core outcome sets for clinical trials: issues to consider, Trials;13:132. https://doi.org/10.1186/1745-6215-13-132

[20] Van Breda AD 2005 Steps to analysing multiple-group NGT data. The Social Work Practitioner-Researcher. 17(1), 1-14.

[21] Ryan M, Bate A, Eastmond C, Ludbrook A 2001 Use of discrete choice experiments to elicit preferences. Quality in Health Care, 10, (Suppl I): i55-i60.

[22] de Bekker-Grob EW, Donkers B, Jonker MF, Stolk EA (2015) Sample Size Requirements for Discrete-Choice Experiments in Healthcare: a Practical Guide Patient, 8: 373-384. https://doi.org/10.1007/s40271-015-0118-z

[23] Coast J, Al-Janabi H, Sutton EJ, Horrocks SA, Vosper AJ, Swancutt DR, Flynn TN, 2012 Using qualitative methods for attribute development for discrete choice experiments: issues and recommendations. Health Economics, 21: 730-741. https://doi.org/10.1002/hec.1739

[24] Soekhai V, de Bekker-Grob EW, Ellis AR, Vass CM 2019 Discrete Choice Experiments in Health Economics: Past, Present and Future. PharmacoEconomis, 37: 201-226. https://doi.org/10.1007/s40273-018-0734-2

[25] de Bekker-Grob EW, Swait JD, Kassahun HT, Bliemer MCJ, Jonker F, Veldwijk J, Cong K, Rose JM, Donkers B 2019 Are Healthcare Choices Predictable? The Impact of Discrete Choice Experiment Designs and Models. Value in Health 22 (9):1050-1062. https://doi.org/10.1016/j.jval.2019.04.1924

[26] Mentzakis E, Ryan M, McNamee P 2011 Using discrete choice experiments to value informal care tasks: exploring preference heterogeneity. Health Economics, 20: 930-944. https://doi.org/10.1002/hec.1656

For peer review only - http://bmjopen.bmj.com/site/about/guidelines.xhtml

[27] Lancsar E, Louviere J 2008 Conducting discrete choice experiments to inform healthcare decision making: a user's guide. PharmacoEconomics, 26 (8):661-677. https://doi.org/10.2165/00019053-200826080-00004

[28] Hole AR 2008 Modelling heterogeneity in patients' preferences for the attributes of a general practitioner appointment. Journal of Health Economics, 27: 1078-1094. https://doi.org/10.1016/j.jhealeco.2007.11.006

[29] O'Cathain A, Murphy E, Nicholl J 2010 Three techniques for integrating data in mixed methods studies. BMJ, 341:c4587. https://doi.org/10.1136/bmj.c4587

[30] T Farmer, K Robinson, SJ Elliott, J Eyles 2006 Developing and implementing a triangulation protocol for qualitative health research. Qualitative Health Research, 16(3):377-394. https://doi.org/10.1177/1049732305285708

For peer review only

Figure 1: Outline of the MOPeD study

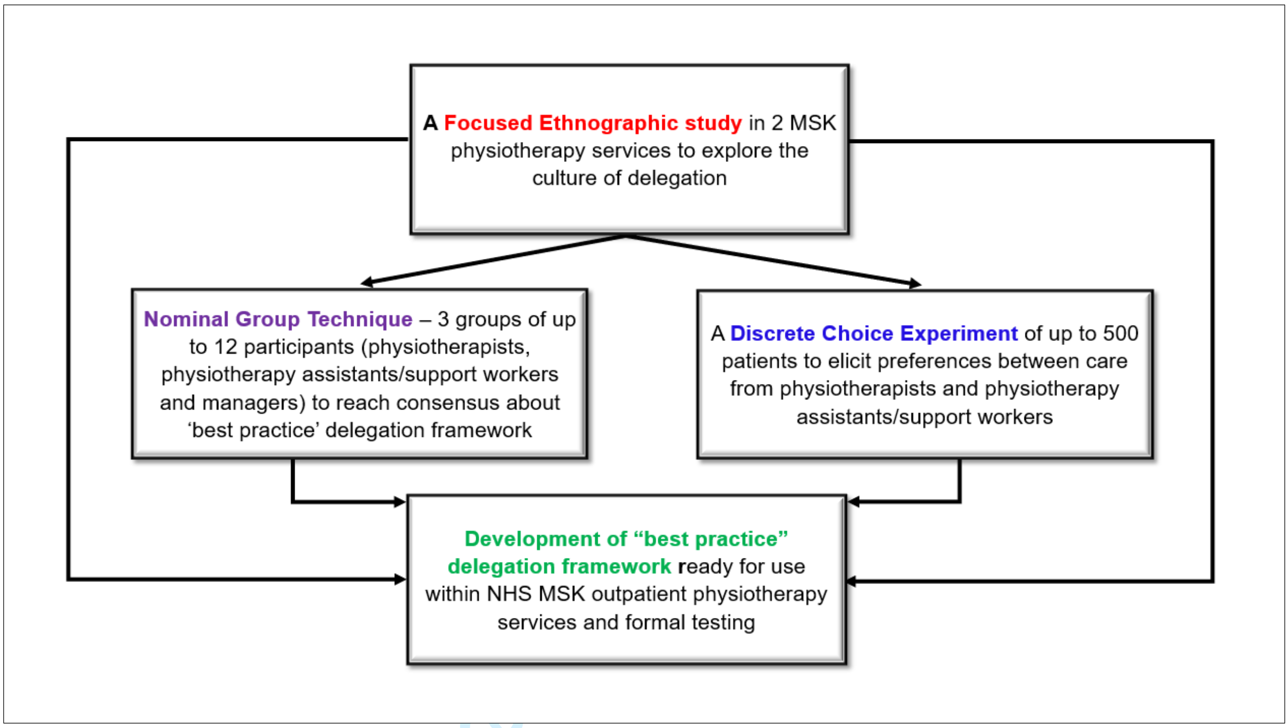

BMJ Open

Developing a best-practice framework for Musculoskeletal Outpatient Physiotherapy Delegation: the MOPeD mixed methods research study protocol

Journal:	*BMJ Open*
Manuscript ID	bmjopen-2023-072989.R1
Article Type:	Protocol
Date Submitted by the Author:	07-Mar-2023
Complete List of Authors:	Sarigiovannis, Panos; Keele University, Primary Care Centre Versus Arthritis, School of Medicine; Midlands Partnership NHS Foundation Trust, North Integrated Musculoskeletal Service<br>Foster, Nadine; The University of Queensland and Metro North Health, STARS Education and Research Alliance, Surgical Treatment and Rehabilitation Service (STARS); Keele University, Primary Care Centre Versus Arthritis, School of Medicine<br>Jowett, Sue; University of Birmingham, Health Economics Unit, Institute of Applied Health Research, Public Health Building<br>Saunders, Benjamin; Keele University, Primary Care Centre Versus Arthritis, School of Medicine
**Primary Subject Heading**:	Evidence based practice
Secondary Subject Heading:	Health services research
Keywords:	Adult orthopaedics < ORTHOPAEDIC & TRAUMA SURGERY, Musculoskeletal disorders < ORTHOPAEDIC & TRAUMA SURGERY, Health & safety < HEALTH SERVICES ADMINISTRATION & MANAGEMENT

SCHOLARONE™
Manuscripts

TITLE

Developing a best-practice framework for Musculoskeletal Outpatient Physiotherapy

Delegation: the MOPeD mixed methods research study protocol

Author(s):

Panos Sarigiovannis, Clinical Doctoral Research Fellow[1,2], Musculoskeletal Clinical Specialist Physiotherapist[2]

Nadine E Foster, Professor and Director, STARS Education and Research Alliance[3], Visiting Professor of Musculoskeletal Health[1]

Sue Jowett, Professor of Health Economics[4]

Benjamin Saunders, Lecturer in Applied Health Research and Deputy Director of the Centre for Musculoskeletal Health Research[1]

Institute(s):

1. Primary Care Centre Versus Arthritis, School of Medicine, Keele University, Staffordshire, ST5 5BG, United Kingdom.
2. Midlands Partnership NHS Foundation Trust, Newcastle under Lyme, Staffordshire, ST5 2BQ, United Kingdom.
3. STARS Education and Research Alliance, Surgical Treatment and Rehabilitation Service (STARS), The University of Queensland and Metro North Health, Herston, Brisbane, Australia.
4. Health Economics Unit, Institute of Applied Health Research, Public Health Building, University of Birmingham, Edgbaston, Birmingham, B15 2TT, United Kingdom.

Authors' email addresses

P Sarigiovannis: p.sarigiovannis1@keele.ac.uk

NE Foster: n.foster@uq.edu.au

S Jowett: s.jowett@bham.ac.uk

B Saunders: b.saunders@keele.ac.uk

Corresponding author:

Sarigiovannis P

ORCID: https://orcid.org/0000-0001-7960-1500

For peer review only - http://bmjopen.bmj.com/site/about/guidelines.xhtml

Address: Primary Care Centre Versus Arthritis, School of Medicine, Keele University, Staffordshire, ST5 5BG, United Kingdom.

Email: p.sarigiovannis1@keele.ac.uk

Twitter: @sarigiovannis1

Word count: 3959

For peer review only

For peer review only - http://bmjopen.bmj.com/site/about/guidelines.xhtml

ABSTRACT

Introduction: Physiotherapy assistants/support workers are an important part of the physiotherapy workforce in the UK. Many of them work in NHS physiotherapy outpatient services treating patients with musculoskeletal (MSK) conditions. In many services they take responsibility, under professional supervision, for types of clinical work traditionally undertaken by physiotherapists such as leading exercise classes and treating individual patients. Nevertheless, their role(s) are relatively undefined and as such there is considerable variation in the duties and tasks they undertake. This study aims to design a framework of 'best practice' in delegation to guide the work of clinicians in NHS physiotherapy MSK services and facilitate standardisation of practice to ensure that patients receive safe and effective treatment by the most appropriate person.

Methods and analysis: This mixed methods study will be conducted in four stages. In stage 1, a focused ethnography in two MSK outpatient physiotherapy services will explore how the current use of delegation is informed by the culture within the clinical setting as well as views, attitudes about, and experiences of, delegation among clinicians, managers and patients. In stage 2a, Nominal Group Technique will be used with three separate groups (physiotherapists/physiotherapy assistants/support workers, managers) to reach consensus about what components should be included in a best practice framework of delegation. In stage 2b, a discrete choice experiment will elicit patients' preferences between care from physiotherapists and physiotherapy assistants/support workers within MSK physiotherapy services. In the final stage, the results of all previous stages will be triangulated to inform the development of a best practice delegation framework for future testing and use within NHS MSK outpatient physiotherapy services.

Ethics and dissemination: Ethical approval has been granted by the South West - Frenchay Research Ethics Committee. The findings will be disseminated in peer-reviewed journals, conference presentations, the lay press and social media.

For peer review only - http://bmjopen.bmj.com/site/about/guidelines.xhtml

Key words: Skill mix, personnel delegation, physiotherapy support workers, mixed methods, protocol

For peer review only

For peer review only - http://bmjopen.bmj.com/site/about/guidelines.xhtml

ARTICLE SUMMARY

Strengths and limitations of this study

- A mixed methods study which includes qualitative and quantitative data and multiple research approaches will enable a more comprehensive understanding of the issues that affect delegation in the MSK physiotherapy setting
- The design of a framework of best practice in delegation to guide the work of physiotherapy assistants/support workers in the MSK physiotherapy setting, underpinned by patients' preferences and clinicians' consensus, may facilitate successful implementation in clinical practice
- The focused ethnographic study is being conducted across two NHS Trusts and the Discrete Choice Experiment across one NHS Trust therefore, the results may not be representative of the NHS more broadly
- Patients and clinicians have been involved in designing the research and they will continue to be involved in all stages of the study

For peer review only

For peer review only - http://bmjopen.bmj.com/site/about/guidelines.xhtml

1 INTRODUCTION

Musculoskeletal (MSK) conditions such as low back pain and osteoarthritis affect one in four people globally, are increasingly common with age, are the leading cause of pain and disability in the UK and the second leading cause of sickness absence from work [1]. Patients with MSK conditions are the largest patient population group treated by physiotherapists [2]. Patients are assessed by physiotherapists and if they need follow-up treatments, they are usually treated by either a physiotherapist or a physiotherapy support worker. Physiotherapy support workers are non-registered staff who work alongside physiotherapists to provide delegated interventions and responsibilities. They may also be known as physiotherapy assistants, rehabilitation assistants, technical instructors or physiotherapy technicians. There are approximately 9,000 physiotherapy assistants/support workers in the UK, forming 15% of the total physiotherapy workforce and a large proportion of them work in the NHS. In many services they take responsibility, under professional supervision, for certain types of clinical work traditionally undertaken by qualified physiotherapists, such as leading exercise classes and treating individual patients. However, their role(s) are relatively undefined and as such, there is considerable variation in the duties and tasks that they undertake [3]. National guidance from the Chartered Society of Physiotherapy (CSP)[1] about delegation of tasks to physiotherapy assistants/support workers largely leave decision-making to the individual physiotherapist, their judgement of the task and their assessment of the competence of the physiotherapy assistant/support worker [4]. As a result, in some physiotherapy services, physiotherapy assistants/support workers have a predominantly clinical role whereas in others they fulfil primarily an administrative role such as data inputting and booking appointments. This latter situation leads to physiotherapy assistants/support workers not being able to utilise their clinical skills, experiencing job dissatisfaction, as well as to unjustified variation in care and clinical services provided to

[1] CSP is the professional, educational and trade union body for the UK's chartered physiotherapists, physiotherapy students and support workers.

For peer review only - http://bmjopen.bmj.com/site/about/guidelines.xhtml

patients [3]. Results from a recent systematic review which explored the clinical and cost-effectiveness and perceptions of delegation by Allied Health Professionals to Allied Health Assistants internationally [5], highlighted that delegation is not standardised within physiotherapy and that there are clear knowledge gaps regarding delegation by physiotherapists in current practice. These relate to the clinical and cost-effectiveness of delegation as well as patients' preferences, experiences of and attitudes about delegation. This study aims to design a framework of best practice in delegation to guide the work of physiotherapy assistants/support workers in NHS physiotherapy MSK services and facilitate standardisation of practice to ensure that patients receive safe and effective treatment by the most appropriate person.

2 RATIONALE

The NHS Long Term Plan includes a commitment to narrow health inequalities and address unwarranted variation in care [6]. A framework of best practice for delegation within the MSK setting could facilitate standardisation of delegation in physiotherapy and therefore, minimise unwarranted variation in the provision of physiotherapy services. Appropriate use of physiotherapy assistants/support workers could reduce healthcare costs either directly or indirectly since it could release capacity for physiotherapists to treat patient cases that are more complex or to be the first point of contact for some patients, in place of a general practitioner (GP) or a consultant doctor. Most importantly, patients would see the right staff with the right skills at the right time, which could potentially optimise clinical outcomes and patient satisfaction. Since staffing costs usually account for between 60% and 80% of operating costs in healthcare services, determining the 'right' combination of staff with the right skills is a critical component of successful and efficient healthcare delivery [7, 8]. The findings of this research will guide workforce planning in MSK physiotherapy services and identify future training needs. Finally, the best practice delegation framework will underpin a

For peer review only

future research study which will assess the clinical and cost-effectiveness of delegation using this framework in clinical settings.

3 THEORETICAL FRAMEWORK

An exploratory sequential mixed methods design will be used. This is characterised by an initial qualitative phase of data collection and analysis followed by a quantitative phase. It also includes a final phase of integration or linking the data from the two separate strands of data. The conceptual framework in the exploratory design will be inductively developed in the initial phase of the study where qualitative data results may lead to a theoretical model [9]. One of the strengths of the exploratory sequential design is that the researcher can produce a new instrument or a framework as one of the potential products of the research process [9].

4 RESEARCH QUESTION/AIM(S)

The overall research question is:

> ‘What should a ‘best practice’ framework of delegation incorporate and how can this be operationalised to guide utilisation of physiotherapy assistants/support workers in NHS MSK outpatient physiotherapy services?’

4.1 Objectives

The specific objectives are to:

1. Explore how the use of delegation is informed by the culture within the clinical setting as well as perceptions of, and attitudes about, delegation among physiotherapists, physiotherapy assistants/support workers, physiotherapy managers and patients.

For peer review only - http://bmjopen.bmj.com/site/about/guidelines.xhtml

2. Reach consensus between physiotherapists, physiotherapy assistants/support workers and physiotherapy managers about what constitutes “best practice” and what components should be included in a best practice framework of delegation in NHS MSK outpatient physiotherapy services.

3. Explore patients’ preferences in relation to delegation in NHS MSK outpatient physiotherapy services and estimate specific trade-offs patients are willing to make in treatment choices when they are treated by physiotherapy assistants/support workers.

4. Develop a best practice delegation framework, which can be tested in future research and used within NHS MSK outpatient physiotherapy services.

An outline of the MOPeD study is shown in figure 1.

[Figure 1 to be inserted here]

For peer review only

For peer review only - http://bmjopen.bmj.com/site/about/guidelines.xhtml

5 METHODS AND ANALYSIS

5.1 Eligibility criteria

The eligibility criteria for each stage of the MOPeD study are shown in table 1.

[Table 1 to be inserted here]

Table 1: Eligibility Criteria for each stage

MOPeD study eligibility criteria	
**Stage 1: Focused ethnography**	1. Adult patients (18 years old or older) with an MSK condition who attend physiotherapy appointments at participating clinics during the period of the study.<br>2. Physiotherapists, physiotherapy assistants/support workers and physiotherapy managers working in participating MSK outpatient physiotherapy services.
**Stage 2a: Consensus study**	1. Experienced physiotherapists (Agenda for Change[2] band 6 or higher) working in the UK NHS in the treatment of MSK conditions.<br>2. Physiotherapy assistants/support workers working in the UK NHS, treating patients with MSK conditions.<br>3. Physiotherapy service managers working in the UK NHS outpatient MSK physiotherapy Services.
**Stage 2b: Discrete choice experiment**	Adult patients (18 years old or older) who have been offered a follow-up physiotherapy appointment and/or completed a course of physiotherapy treatment for a MSK condition in one of the MSK outpatient physiotherapy clinics within the participating NHS Trust.

5.2 Stage 1

5.2.1 Design: A focused ethnographic study

A focused ethnography will be conducted in two MSK outpatient physiotherapy services.

Focused ethnography explores a specific issue, situation or problem within a specific context

[2] Physiotherapists working in the NHS are employed under the Agenda for Change (AfC) grading and pay system where higher bandings are associated with higher qualifications and pay. Newly qualified physiotherapists work in Band 5 positions, while physiotherapists who have completed their junior rotations are in band 6 positions.

For peer review only - http://bmjopen.bmj.com/site/about/guidelines.xhtml

[10, 11]. It is very suitable for healthcare research as it provides an efficient way to capture in-depth data on a specific topic of importance to individual clinicians or clinical specialities, and to determine ways to improve care and care processes [12]. This study has a clear focus on delegation, in a specific clinical setting and the research team has prior experience of using and organising clinical delegation. Therefore, focused ethnography will enable the researchers to explore how the use of delegation is informed by the culture of two different clinical settings, which will allow for the development of an in-depth and comparative understanding about how delegation is currently being used, and the factors that influence delegation to physiotherapy assistants/support workers at an individual, collective, and broader organisation level. It will also allow the researchers to better understand attitudes about, and experiences of, delegation among physiotherapists, physiotherapy assistants/support workers, physiotherapy managers and patients.

5.2.2 Data collection

Stage 1/Focused ethnography

A purposive sampling design has been selected as two physiotherapy services that are known to use delegation successfully (positive deviance approach) will be observed. These were chosen in collaboration with the appropriate professional advisor from the CSP who currently leads the support workers' workstream, taking into consideration different indicators such as continuous use of physiotherapy assistants/support workers in treating patients with MSK conditions and dissemination of clinical practice. Within stage 1, criterion sampling will be used as the purposeful sampling design for the selection of patients who will be invited to participate in the observations and/or interviews as only patients who have been treated by a physiotherapist and a physiotherapy assistant/support worker will be invited.

The researcher will use participant observation in two clinical sites focusing on current delegation practice, semi-structured interviews with physiotherapists, physiotherapy

For peer review only - http://bmjopen.bmj.com/site/about/guidelines.xhtml

assistants/support workers, managers and patients, as well as a review of physiotherapy treatment records and clinical site records such as policies, job descriptions and delegation training records to gain a rich and comprehensive understanding of how delegation works in the observed setting. Whenever appropriate, similarities and differences across the different types of data will be explored. Field observation will involve observing a range of activities including clinical sessions (one to one or groups) that physiotherapy assistants/support workers deliver independently or participate in, supervision sessions, training sessions and team meetings. Field notes will include information about observed events but also the researcher's personal reflections and interpretation of events [13].

All physiotherapy assistants/support workers who work in the observed clinical setting, the physiotherapists who delegate clinical tasks to them, the operational manager(s)/team leader(s) and patients will be invited to participate in the interviews. Up to 12 patients and 12 clinicians (physiotherapists, physiotherapy assistants/support workers, manager/s) will be interviewed at each site (i.e. up to 24 interviews in total per site). A final decision on sample size will be made once data collection and analyses are ongoing, based on data saturation [14].

The interview topic guides have been developed based on the evidence from a systematic review conducted by Sarigiovannis et al [5]. An example of the interview topic guides is included in appendix 1. The interviews will give the researcher the opportunity to ask physiotherapists, physiotherapy assistants/support workers, managers, and patients for elaborations about specific topics, explanations of observed events and clarification of ambiguities [15]. Questions will cover topics such as how the tasks were delegated, how patients feel about the delegation of clinical tasks etc. The questions have been reviewed by the study's Patient and Public Involvement and Engagement (PPIE) and Clinical Advisory Groups to ensure acceptability of the questions and ease of understanding. The examination of relevant documents such as patients' physiotherapy treatment records and relevant policies will help validate the data from observations and interviews.

For peer review only - http://bmjopen.bmj.com/site/about/guidelines.xhtml

5.2.3 Data analysis

Data analysis will be based on the approach described by Roper and Shapira [15]. Analytical steps include coding field notes and interviews, sorting to identify patterns for descriptive labels, identifying outliers or cases that do not "fit" with the rest of the findings, generalising in relation to existing concepts and theories as well as the ideas and insights the researcher has about the data, including their reflective field notes [15]. The analytical steps will be focused on answering specific problem-orientated research questions and the creation of concrete recommendations. The qualitative data analysis software QSR NVivo will be used to facilitate analysis. Data collection for stage 1 started in March 2022. The data analysis will be completed by June 2023.

5.3 Stage 2a

5.3.1 Design: A Nominal Group Technique (NGT) approach

A consensus study will be carried out, using Nominal Group Technique (NGT) a systematic approach to building consensus using a structured set of stages. A convenience sample of physiotherapists, physiotherapy assistants/support workers and physiotherapy managers who work in NHS MSK Physiotherapy Services will be recruited through the CSP's professional networks and through social media (Facebook, Twitter and LinkedIn) accounts of the authors and their networks. Three separate role-specific NGT working groups will be convened, with; 1) physiotherapists; 2) managers; and 3) physiotherapy assistants/support workers with the aim to reach agreement about what a 'best practice' delegation framework should include. Each participant group will take part in a single meeting lasting approximately two hours. Two facilitators will lead each meeting. They will use a 'pre-elicitation technique' to inform the participants' decision-making [16]. Specifically, prior to the meeting participants will receive a summary of existing evidence based on the findings from a systematic review

For peer review only - http://bmjopen.bmj.com/site/about/guidelines.xhtml

conducted by Sarigiovannis et al [5]. The participants will initially be asked to silently generate ideas about what should be included in a delegation framework for use within the MSK physiotherapy setting, which will then be shared with the group one by one. Each shared item will then be individually rated by all participants. Following this, the results of these ratings will be discussed within the group, and then they will be re-rated individually by participants, who will be given the opportunity to amend their scores in light of group discussions.

The rating process will be explained step-by-step and participants will be given enough time for voting [17]. Each item will be rated on a 7-point Likert scale by individual participants and a mean rating calculated. The threshold for consensus will be set at ≥70% i.e. ≥ 4.9 on the 7-point Likert scale across the group [18,19]. The data from the 3 groups will be combined into one complete sample to identify the most highly prioritised items [20]. This includes calculating the mean scores for the raw data, the mean scores for the themes and the frequency of themes (i.e. how many times a theme appeared in the top five and how often an idea was raised and coded under the same theme). All items will be categorised using a modified thematic analysis approach which will include generating initial themes from the collated data and refining themes. The items will be rated using an online voting platform called Mentimeter (https://www.mentimeter.com/) which participants will access on their smartphones or laptops. The issues discussed during the meeting will not include any topics that might be sensitive, embarrassing or upsetting. The facilitators will keep notes during the consensus meetings. A list of all items that reached consensus categorised into themes will be the final output from the consensus study.

5.4 Stage 2b

5.4.1 Design: A Discrete Choice Experiment (DCE)

For peer review only - http://bmjopen.bmj.com/site/about/guidelines.xhtml

Discrete choice experiments (DCEs) are an attribute-based survey method for measuring benefits (utility). Within healthcare, the technique is applied to address a wide range of issues in the delivery of healthcare including measuring and valuing attributes of a healthcare service and identifying the factors that influence choices and decisions of patients, the public and healthcare professionals [21]. They are based on the assumption that a service can be described by its characteristics or attributes, and the extent to which an individual values the service depends on the levels of these characteristics [21]. In a DCE, respondents are asked to choose between two or more choice sets.

A DCE will be designed based on the data from the ethnography as well as further input from the study's Clinical Advisory Group and PPIE group. The DCE will be conducted to elicit patients' preferences about the use of delegation to physiotherapy assistants/support workers within MSK physiotherapy services. Convenience sampling will be used during this stage to invite patients who have been offered a follow up physiotherapy appointment and/or completed a course of physiotherapy treatment programme in one of the MSK outpatient physiotherapy services within the participating NHS Trust. Patients will be invited by the treating clinicians when patients attend their follow up physiotherapy appointment/are discharged. The minimum sample size needed for the DCE depends on the specific hypotheses to be tested [22]. Therefore, the power of the DCE will be calculated when the questionnaire is finalised. It is expected that the number of participants will not exceed 500. This number is feasible since approximately 1500 patients are discharged per month within the Trust.

5.4.2 Data collection

Development of the attributes and levels will be undertaken using the appropriate findings from a systematic review on delegation by Allied Health Professionals (AHPs) to support workers [5]; the ethnographic data from stage 1 of the study; and further input from the study's PPIE group [23]. A recent systematic review of DCEs in healthcare reported that most studies included four to nine attributes, and among them four to five was the modal

For peer review only - http://bmjopen.bmj.com/site/about/guidelines.xhtml

category [24]. The intention is to select between five to seven attributes. The Ngene design software will be used to create the choice sets. In addition to the DCE, the survey will include items to elicit sociodemographic information such as age, gender, and employment status as well as experience of being treated by a physiotherapy assistant/support worker. These seem to play an important role in predicting healthcare choices [25].

The DCE survey will be offered in an online format, completed in physiotherapy clinics using tablet devices. The online version will be saved on a secure server. The understanding of the attributes and levels will be pretested among members of the PPIE group. The survey will then be piloted with a small sample of patients with MSK conditions consulting physiotherapy services. This will include testing respondent understanding of the different choices offered, generation and testing of appropriateness and understanding of attributes/levels, task complexity, length, timing and likely response rates [26, 27].

For peer review only

5.4.3 Data analysis

Data analysis will be completed using the STATA software. Although preference heterogeneity has long been accounted for in the analysis of DCEs by interacting design attributes with socio-demographic characteristics, evidence suggests that this approach only partially accounts for the differences in preference embodied in the data [28]. Research has shown that DCE models that take into account both preference heterogeneity and differences in the error variance of choices (scale heterogeneity) are better to predict choices mimicking real-world decisions [23]. Therefore, selecting the appropriate model for data analysis is important. The choice of the model will be finalised following discussions with DCE experts. The results will help the researchers understand what influences patients' preferences by estimating the value patients place on key attributes and associated attribute levels. The researchers will estimate specific trade-offs that patients who are treated in NHS MSK outpatient physiotherapy services are willing to make in treatment choices, specifically in relation to being treated by physiotherapy assistants/support workers.

For peer review only - http://bmjopen.bmj.com/site/about/guidelines.xhtml

5.5 Stage 3

5.5.1 Design and analysis: Development of a best practice framework of delegation

The results from stages 1, 2a and 2b will be triangulated to inform the design of a best-practice delegation framework for NHS MSK outpatient physiotherapy services. Triangulation will involve listing the findings from each stage of the study and consider where findings from each stage agree (convergence), offer complementary information on the same issue (complementarity), or appear to contradict each other (discrepancy or dissonance) [29].

Triangulation of findings will be conducted based on Farmer et al's triangulation protocol [30]. This involves identifying themes from each stage of the study and then sorting them into similar categories. The themes will be "convergence coded" to identify where there is agreement, silence and dissonance in terms of data from the different stages of the study [30]. This technique for triangulation is the only one to include silence or where a theme or finding arises from one data set and not another. Silence might be expected because of the strengths of each method to examine different aspects of delegation, but surprise silences might also arise that help to increase understanding [29]. The format of the framework e.g. paper document or online tool, will be guided by data from stages 1 and 2a and consultation with the Clinical Advisory and PPIE groups. This will then be formally tested in post-doctoral research. It is anticipated that stage 3 will be completed by June 2024.

5.6 Patient and Public Involvement and Engagement

Patients and/or the public were/will be involved in the design, or conduct, or reporting, or dissemination plans of this research. The researchers have worked with a group of seven patients to develop this mixed methods research study protocol. They all have experience of

For peer review only - http://bmjopen.bmj.com/site/about/guidelines.xhtml

treatment by physiotherapists and/or physiotherapy assistants/support workers for a MSK condition.

The PPIE members have reviewed the patient participant information leaflets and consent forms associated with this protocol. The researchers will continue working with the PPIE group to:

- Produce/amend the appropriate materials to inform participants and the public about the study
- Analyse/interpret the data from the patient interviews and the DCE
- Design the best practice framework of delegation
- Produce the materials for sharing the results publicly and decide where to share the results

5.7 Clinical Advisory Group

A group of clinicians consisting of four physiotherapists, three physiotherapy assistants/support workers, one physiotherapy manager, one clinical lead, and a Professional Advisor from the CSP have helped shape the research plans. The group will continue providing support to this study including supporting its delivery, interpretation of results and dissemination.

For peer review only - http://bmjopen.bmj.com/site/about/guidelines.xhtml

6 ETHICS AND DISSEMINATION

Written informed consent will be obtained prior to the participants undergoing any activities that are specifically for the purposes of the study. The study requires ethical approval for stages 1, 2a and 2b. Approvals have been received from the South West - Frenchay Research Ethics Committee (17 December 2021 IRAS ID: 297095, REC Reference 21/SW/0158 & 29 December 2022, Amendment number AM02 SA01).

Publications reporting on each stage of the study will be prepared for peer-reviewed open access journals. Additionally, abstracts will be submitted for presentation at local, national and international conferences such as Physiotherapy UK, Health Services Research UK and the World Confederation for Physical Therapy Congress. The results will be shared with the appropriate professional groups and networks within the CSP and at participating sites through in-service-training. A webinar will be prepared for physiotherapists and physiotherapy assistants/support workers and will be shared via the CSP's website and on social media. Furthermore, the results will be presented at local dissemination events that will be organised in each participating Trust involving all key stakeholders. Finally, a plain English summary of the results will be shared with participants and the public via social media, selected magazines and/or newspapers as well as MSK patient groups via Versus Arthritis or other charities.

For peer review only

For peer review only - http://bmjopen.bmj.com/site/about/guidelines.xhtml

Author Contributions

PS wrote the paper and conceived the study with the support of NEF, SJ and BS. All authors have read, edited and approved the final version.

Funding

Panos Sarigiovannis was funded by National Institute for Health and Care Research awards (HEE/NIHR ICA Programme Clinical Doctoral Research Fellowship NIHR301550) for this research project.

Disclaimer

The views expressed in this publication are those of the author(s) and not necessarily those of the NIHR, NHS or the UK Department of Health and Social Care.

Competing interests

None declared.

Data availability statement

No additional data available.

For peer review only

REFERENCES

[1] Versus Arthritis 2019 The State of Musculoskeletal Health. Arthritis and other Musculoskeletal conditions in number https://www.versusarthritis.org/media/14594/state-of-musculoskeletal-health-2019.pdf [Accessed on 18/06/2021]

[2] The Chartered Society of Physiotherapy (CSP) 2013 Physiotherapy Works: Musculoskeletal Disorders, https://www.csp.org.uk/system/files/csp_physiotherapy_works_msk_june_2013.pdf [Accessed on 18/06/2021]

[3] Sarigiovannis P; Cropper S 2018 An audit of the utilization of physiotherapy assistants in the musculoskeletal outpatients setting within a primary care physiotherapy service. Musculoskeletal Care, 16(3):405–408 https://doi.org/10.1002/msc.1238

[4] The Chartered Society of Physiotherapy (CSP) 2017 Information Paper PD126 Supervision, Accountability & Delegation, https://www.csp.org.uk/system/files/supervision_accountability_delegation_final.pdf [Accessed on 18/06/2021]

[5] Sarigiovannis P, Jowett S, Saunders B, Corp N, Bishop A 2021 Delegation by Allied Health Professionals to Allied Health Assistants: a mixed methods systematic review. Physiotherapy, 112:16-30. https://doi.org/10.1016/j.physio.2020.10.002

[6] NHS England 2019 The NHS Long Term Plan. https://www.longtermplan.nhs.uk/wp-content/uploads/2019/08/nhs-long-term-plan-version-1.2.pdf [Accessed on 18/06/2021]

[7] Buchan J, Dal Poz MR 2002 Skill mix in the health care workforce: reviewing the evidence. Bulletin of the World Health Organisation, 80(7):575-580. https://www.who.int/hrh/documents/skill_mix.pdf [Accessed on 18/06/2021]

[8] Nancarrow SA, Borthwick AM 2005 Dynamic professional boundaries in the healthcare workforce. Sociology of Health & Illness, 27(7):897-919. https://doi.org/10.1111/j.1467-9566.2005.00463.x

[9] Creswell JW, Plano Clark VL 2018 Designing and conducting mixed methods research. International student edition (Third edition). Sage Publications, Los Angeles.

[10] Rashid M, Hodgson CS, Luig T 2019 Ten tips for conducting focused ethnography in medical education research. Medical Education Online, 24(1):1624133 https://doi.org/10.1080/10872981.2019.1624133

[11] Knoblauch H 2005 Focused Ethnography. Forum Qualitative Social Research (FQS); 6 (3):Art. 44 https://doi.org/10.17169/fqs-6.3.20

For peer review only - http://bmjopen.bmj.com/site/about/guidelines.xhtml

[12] Higginbottom GMA, Pillay JJ, Boadu NY 2013 Guidance on Performing Focused Ethnographies with an Emphasis on Healthcare Research. The Qualitative Report, 18, Article 17:1-16. https://doi.org/10.46743/2160-3715/2013.1550

[13] Finlay L 2002 "Outing" the Researcher: The Provenance, Process, and Practice of Reflexivity Qualitative Health Research, 12 (4):531-545. https://doi.org/10.1177/104973202129120052

[14] Saunders B, Sim J, Kingstone T, Baker S, Waterfield J, Bartlam B, Burroughs H, Jinks C 2018 Saturation in qualitative research: exploring its conceptualization and operationalization. Quality & Quantity 52 (4):1893-1907. https://doi.org/10.1007/s11135-017-0574-8

[15] Roper JM, Shapira J 2000 Ethnography in Nursing Research. Sage Publications, Thousand Oaks, California.

[16] Protheroe J, Saunders, B., Bartlam, B, Dunn KM, Cooper V, Campbell P, Hill JC, Tooth S, Mallen CD, Hay EM, Foster NE 2019 Matching treatment options for risk sub-groups in musculoskeletal pain: a consensus groups study. BMC Musculoskeletal Disorders, 20: 271, https://doi.org/10.1186/s12891-019-2587-z

[17] McMillan SS, Kelly F, Sav A, Kendall E, King MA, Whitty JA, Wheeler AJ 2014 Using the Nominal Group Technique: how to analyse across multiple groups. Health Services and Outcomes Research Methodology, 14:92-108. https://doi.org/10.1007/s10742-014-0121-1

[18] List D 2001 The Consensus Group Technique in Social Research. Field Methods, 13 (3): 277–290. https://doi.org/10.1177/1525822X0101300304

[19] PR Williamson DG Altman, JM Blazeby, M Clarke, D Devane, E Gargon, P Tugwell 2012 Developing core outcome sets for clinical trials: issues to consider, Trials;13:132. https://doi.org/10.1186/1745-6215-13-132

[20] Van Breda AD 2005 Steps to analysing multiple-group NGT data. The Social Work Practitioner-Researcher. 17(1), 1-14.

[21] Ryan M, Bate A, Eastmond C, Ludbrook A 2001 Use of discrete choice experiments to elicit preferences. Quality in Health Care, 10, (Suppl I): i55-i60.

[22] de Bekker-Grob EW, Donkers B, Jonker MF, Stolk EA (2015) Sample Size Requirements for Discrete-Choice Experiments in Healthcare: a Practical Guide Patient, 8: 373-384. https://doi.org/10.1007/s40271-015-0118-z

[23] Coast J, Al-Janabi H, Sutton EJ, Horrocks SA, Vosper AJ, Swancutt DR, Flynn TN, 2012 Using qualitative methods for attribute development for discrete choice experiments: issues and recommendations. Health Economics, 21: 730-741. https://doi.org/10.1002/hec.1739

[24] Soekhai V, de Bekker-Grob EW, Ellis AR, Vass CM 2019 Discrete Choice Experiments in Health Economics: Past, Present and Future. PharmacoEconomis, 37: 201-226. https://doi.org/10.1007/s40273-018-0734-2

[25] de Bekker-Grob EW, Swait JD, Kassahun HT, Bliemer MCJ, Jonker F, Veldwijk J, Cong K, Rose JM, Donkers B 2019 Are Healthcare Choices Predictable? The Impact of Discrete

For peer review only

Choice Experiment Designs and Models. Value in Health 22 (9):1050-1062. https://doi.org/10.1016/j.jval.2019.04.1924

[26] Mentzakis E, Ryan M, McNamee P 2011 Using discrete choice experiments to value informal care tasks: exploring preference heterogeneity. Health Economics, 20: 930-944. https://doi.org/10.1002/hec.1656

[27] Lancsar E, Louviere J 2008 Conducting discrete choice experiments to inform healthcare decision making: a user's guide. PharmacoEconomics, 26 (8):661-677. https://doi.org/10.2165/00019053-200826080-00004

[28] Hole AR 2008 Modelling heterogeneity in patients' preferences for the attributes of a general practitioner appointment. Journal of Health Economics, 27: 1078-1094. https://doi.org/10.1016/j.jhealeco.2007.11.006

[29] O'Cathain A, Murphy E, Nicholl J 2010 Three techniques for integrating data in mixed methods studies. BMJ, 341:c4587. https://doi.org/10.1136/bmj.c4587

[30] T Farmer, K Robinson, SJ Elliott, J Eyles 2006 Developing and implementing a triangulation protocol for qualitative health research. Qualitative Health Research, 16(3):377-394. https://doi.org/10.1177/1049732305285708

For peer review only

For peer review only - http://bmjopen.bmj.com/site/about/guidelines.xhtml

LIST OF FIGURES

Figure 1: Outline of the MOPeD study

APPENDICES

Appendix 1: Interview topic guide

For peer review only

For peer review only - http://bmjopen.bmj.com/site/about/guidelines.xhtml

Figure 1: Outline of the MOPeD study

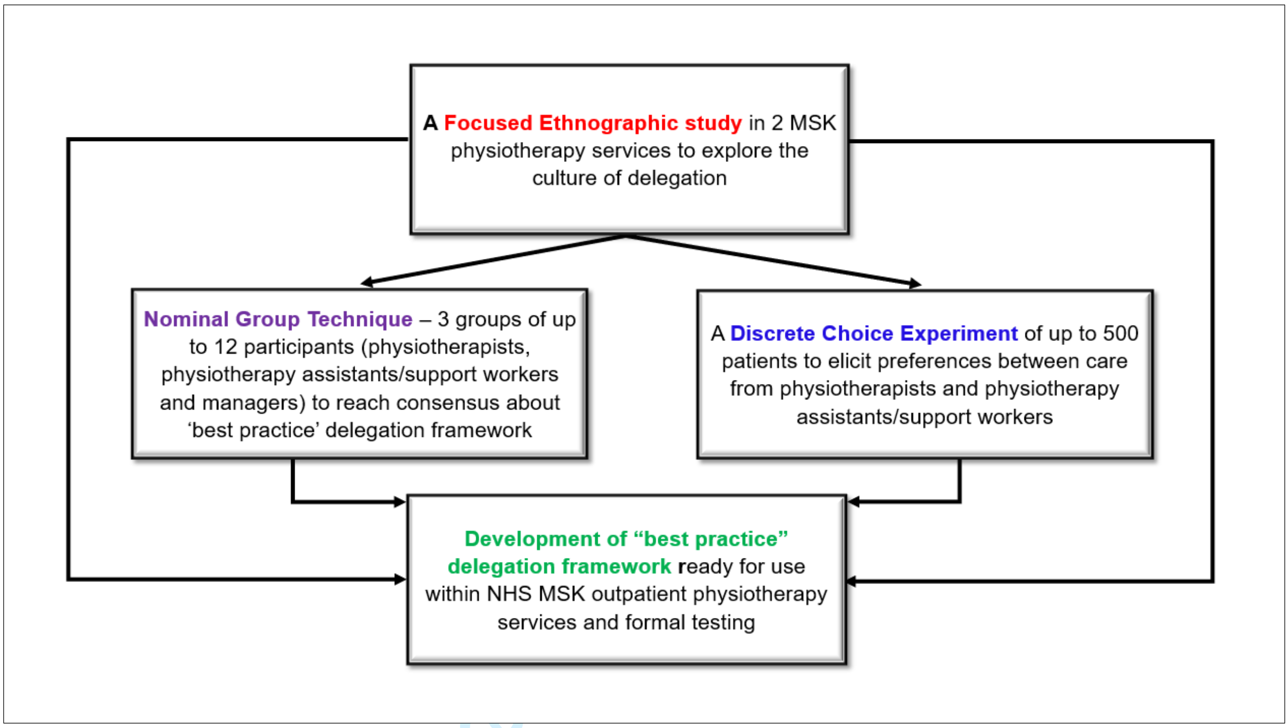

Musculoskeletal Outpatient Physiotherapy Delegation (MOPeD) (Stage 1, Focused Ethnography)

Topic Guide: Physiotherapists

1. Introduction

Check that participant has read and understood the Participant Information Leaflet (patient version)
Explain arrangements for: consent, recording, anonymity, expenses where appropriate etc.

2. Training on delegating clinical tasks to physiotherapy assistants (where possible invite participants to expand on their responses)

1) Can you tell me about your experience as a physiotherapist i.e. how many years have you worked as a physio?
2) How many years have you worked in the MSK setting?
3) Have you had any formal or informal training in relation to delegating clinical tasks to physiotherapy assistants? Probe for types of training e.g. formal/informal, when etc.

3. Experience and acceptability of delegating tasks to physiotherapy assistants (where possible invite participants to expand on their responses)

4) Do you always delegate clinical tasks to physiotherapy assistants?
5) Is this a personal choice or do you follow agreed treatment pathways?
6) Are you aware of any professional guidance about delegation? If yes, can you name it?
7) How do you feel about delegating part of your clinical treatment? If yes/no, can you explain why?
8) Are there any parts of the treatment plan that you do not delegate? Why?
9) Can you talk about a complete treatment where delegation worked really well? Why did it work well?
10) Can you recall a case where delegation did not work so well? Why do you think this happened?
11) How do you feel about delegating clinical tasks to physiotherapy assistants?

4. Close of discussion

a. Summary of discussion: any additional remarks?
b. Check consent is still in place.
c. Check if participant would like to receive a summary of the interview findings.

For peer review only - http://bmjopen.bmj.com/site/about/guidelines.xhtml
