# Supplementary material for: Developing a best practice framework for musculoskeletal outpatient physiotherapy delegation: the MOPeD mixed-methods research study protocol
Source: BMJ Open. 2023 Mar 17;13(3):e072989. doi: 10.1136/bmjopen-2023-072989 (PMC10030620; doi:10.1136/bmjopen-2023-072989)
Supplement: Reviewer comments [file bmjopen-2023-072989.reviewer_comments.pdf]

PEER REVIEW HISTORY

BMJ Open publishes all reviews undertaken for accepted manuscripts. Reviewers are asked to complete a checklist review form (**http://bmjopen.bmj.com/site/about/resources/checklist.pdf**) and are provided with free text boxes to elaborate on their assessment. These free text comments are reproduced below.

ARTICLE DETAILS

**TITLE (PROVISIONAL)**	Developing a best-practice framework for Musculoskeletal Outpatient Physiotherapy Delegation: the MOPeD mixed methods research study protocol
**AUTHORS**	Sarigiovannis, Panos; Foster, Nadine; Jowett, Sue; Saunders, Benjamin

This article was not externally reviewed at BMJ Open. Protocols that have been independently assessed prior to submission to BMJ Open are usually fast-tracked to publication on the grounds that further substantial changes will not be possible. This independent assessment will usually be external, independent review for both a substantial grant award from a non-commercial or government funder and ethics approval.
